# Functional Characterization of a Juvenile Hormone Esterase Related Gene in the Moth *Sesamia nonagrioides* through RNA Interference

**DOI:** 10.1371/journal.pone.0073834

**Published:** 2013-09-11

**Authors:** Dimitrios Kontogiannatos, Luc Swevers, Katsumi Maenaka, Enoch Y. Park, Kostas Iatrou, Anna Kourti

**Affiliations:** 1 Laboratory of Molecular Biology, Department of Agricultural Biotechnology, Agricultural University of Athens, Athens, Greece; 2 Insect Molecular Genetics and Biotechnology Group, Institute of Biosciences & Applications, National Centre for Scientific Research “Demokritos”, Athens, Greece; 3 Department of Biomolecular Chemistry, Graduate School of Pharmaceutical Sciences, Hokkaido University, Kita-ku, Sapporo, Japan; 4 Laboratory of Biotechnology, Department of Applied Biological Chemistry, Faculty of Agriculture, Shizuoka University, Suruga-ku, Shizuoka, Japan; CNRS, France

## Abstract

Juvenile hormone esterase (JHE) is a carboxylesterase that has attracted great interest because of its critical role in regulating larval to adult transition in insects and other arthropods. Previously, we characterized an ecdysteroid sensitive and juvenile hormone non-susceptible juvenile hormone esterase related gene (*SnJHER*) in the corn stalk borer, *Sesamia nonagrioides*. *SnJHER* was rhythmically up-regulated close to each molt during the corn stalk borer’s larval development. In this paper we attempted to functionally characterize *SnJHER* using several reverse genetics techniques. To functionally characterize *SnJHER*, we experimented with different dsRNA administration methods, including hemolymph, bacterial or baculovirus-mediated RNA interference, (RNAi). Our findings indicate the potential implication of *SnJHER* in the developmental programming of *Sesamia nonagrioides*. It is still unclear whether *SnJHER* is closely related to the authentic *JHE* gene, with different or similar biological functions.

## Introduction

Carboxylesterases (COEs) are a multifunctional superfamily ubiquitous in all living organisms [1]. Insect COEs have been the subject of intense research, in terms of their catalytic mechanism, molecular evolution and developmental regulation [2]. Based on sequence similarity and substrate specificity, insect COE genes can be subdivided into eight subfamilies: a-esterases, b-esterases, juvenile hormone esterases, gliotactins, acetylcholinesterases, neurotactins, neuroligins and glutactin class [1]. Lepidopteran insects have been known to possess a high number of COEs in their genome, sometimes making extremely difficult to distinguish them in terms of substrate specificity and biological function. For instance, in the cotton bollworm, *Helicoverpa armigera*, 39 putative carboxyl/cholinesterases (CCEs) sequences have been found scattering in its genome [3], while in the silkworm *Bombyx mori* the total amount of the putative CCEs genes is equivalent to 69 [4].

Juvenile hormone esterase (JHE) is a COE that has attracted great interest for its critical role in regulating larval to adult transition in insects and other arthropods. JHE hydrolyzes the key developmental and reproductive hormone, juvenile hormone (JH) and partially regulates its titer [5], [6], [7]. Juvenile hormone (JH) plays a major role in the control of growth, development, metamorphosis, diapause and reproduction in insects [8], [9]. The onset of metamorphosis is preceded by a decrease in the biosynthesis of JH and an increase in JHE activity [7]. This then sets the stage for the elevation of ecdysteroid titer [10]. JH is normally present at the time of increase in ecdysteroid titers for larval molts and ensures that larvae molt to the next larval stage. However, at the time of the final larval molt, JH disappears allowing ecdysone to induce metamorphosis [11]. JHE is crucial for JH hemolymph titer reduction and therefore the initiation of metamorphosis in diverse insects. Strong inhibition of JHE activity in *S. nonagrioides* larvae has no effect on the onset of metamorphosis [12]. The transcripts of JHE-encoding genes that have already been described in insects are strongly induced by JH, e.g. *Trichoplusia ni* [13], *Heliothis virescens* [14], *Leptinotarsa decemlineata* [15], *Choristineura fumiferana* [16], *Bombyx mori* [17], *Drosophila melanogaster* [18] and *Nilaparvata lugens* [19].

In previous studies we characterized an ecdysteroid sensitive and juvenile hormone non-susceptible juvenile hormone esterase-related gene (*SnJHER*) in the moth *Sesamia nonagrioides* (Lefebvre) (Lepidoptera: Noctuidae) [20]. SnJHER has all the typical motifs of JHEs (RF, DQ, E, GxxHxxD/E). The primary structure of the deduced amino acid sequence of the cDNA showed that the catalytic site of SnJHER has a cysteine residue next to the catalytic serine (GQSCG), while most described juvenile hormone esterases have alanine at this position (GQSAG). The JH analog methoprene did not affect *SnJHER* gene expression, whereas ecdysteroids and xenobiotics induced it. *SnJHER* mRNAs reached higher expression levels on the days close to each larval molt.

The corn stalk borer, *S. nonagrioides*, is a multivoltine species that causes significant damage on maize throughout the Mediterranean basin. Larvae that develop under long-day conditions invariably pupate at the end of the 6^th^ larval instar, while those grown under a short-day photoperiod enter diapause and undergo several supernumerary larval molts. Corn borer larvae programmed for diapause increase their body weights continuously until the 9^th^ instar [20].

RNA interference (RNAi) is a valuable tool for reverse functional genomics. In genetically transformable species, RNAi can be triggered by expressing long double stranded hairpin RNAs in the transformed cells and tissues [21] while in non-model insect species, RNAi can be triggered by delivering *in vitro* synthesized dsRNAs to a chosen stage (from egg to adult) and then examining the resulting phenotype [21]. Moreover in insects, RNAi can be induced via the oral route, either by feeding them directly with *in vitro* synthesized dsRNAs or with bacteria expressing the dsRNAs *in vivo* [22]. In comparison with producing dsRNAs *in vitro*, bacterially expressed dsRNAs is a low cost method and is more easily used in large scale gene function analysis [22]. In addition to the above RNAi techniques, RNAi can be triggered either by infecting insects with recombinant baculoviruses [23] or other viruses [24] that express the dsRNAs in the infected cells.

In this study we examined the functional role of *SnJHER* in the regulation of the corn stalk borer’s larval, pupal and adult development, using several reverse genetics approaches. The dsRNAs were delivered indirectly by using either baculovirus or bacterial vectors or directly after hemolymph administration. Moreover, we investigated the relative capacity of each one of these techniques to induce a *SnJHER* RNAi (SnJHERi) specific phenotype. We conclude that *SnJHER* is implicated in the developmental programming of *S. nonagrioides*, however the exact mechanism of this regulation it is still unknown. Further biochemical and molecular data, are required in order to further elucidate the function of this particular esterase gene with key roles in the developmental regulation of *S. nonagrioides*.

## Results

### RNA Silencing

#### Hemolymph dsRNA administration

**Figure 1:**
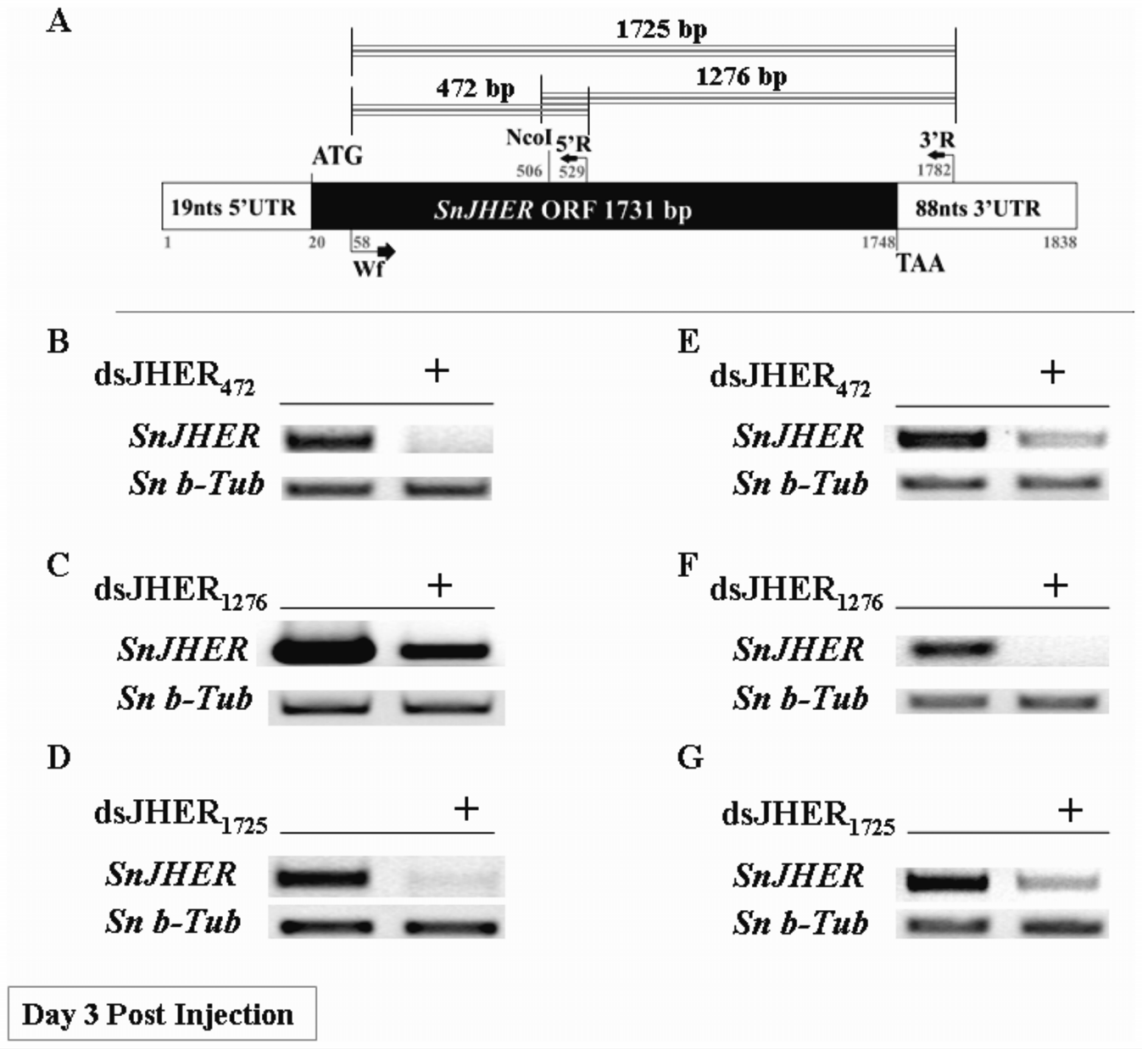
Targeting the *SnJHER* 472/1276/1725 bp part after hemolymph injection of L5d3 and L6d9 larvae. **A.** Schematic representation of *SnJHER* gene. The black color represents the *SnJHER* ORF, while the white color represents the 5′ and 3′ untranslated regions of the gene. **B., E.** Semiquantitative RT-PCR analysis of *SnJHER* mRNA levels, of a randomly selected individual injected with the dsJHER_472_ as 5^th^ instar d3 (B.) or 6^th^ instar d9 larva (E.) and its randomly selected control 3 days post injection. **C., F.** Semiquantitative RT-PCR analysis of *SnJHER* mRNA levels, of a randomly selected individual injected with the dsJHER_1276_ as 5^th^ instar d3 (C.) or 6^th^ instar d9 larva (F.) and its randomly selected control 3 days post injection. **D., G.** Semiquantitative RT-PCR analysis of *SnJHER* mRNA levels, of a randomly selected individual injected with the dsJHER_1725_ as 5^th^ instar d3 (D.) or 6^th^ instar d9 larva (G.) and its randomly selected control 3 days post injection.

For larval and prepupal stages we injected animals with specific dsRNAs which target three different regions of *SnJHER* cDNA, a 472 bp part of its 5′-translated region (Fig. 1A), a 1276 bp part of its central translated, 3′-translated and part of its 3′ -untranslated region (Fig. 1A) and a 1725 bp part encompassing both of the above regions, which spans 94% of the total cDNA (Fig. 1A). The experiments were performed in independent triplicates (three trials) of a total amount of 100 insects. Each trial consisted of at least 30 insects either for control and experimental groups (Table S2, S3, S4 and S5 in File S1). For RT-PCR analyses, we randomly selected 15 insects of each treatment and replicate, 3 days post injection. These were analyzed as individuals and subjected in semi-quantitative RT-PCR analyses in order to measure the *SnJHER* mRNA levels (Table S1 in File S1). Some examples of SnJHERi analyzed individuals are presented in Fig. 1B–1G.

For larval stage we injected animals of 5^th^ instar d3, in which the *SnJHER* mRNAs were higher comparing to the other larval stages [20]. Targeting the 472 bp part of the 5′-translated region of *SnJHER* resulted in a decrease of *SnJHER* mRNA levels (Fig. 1B). In contrast to the transcriptional effect, no phenotypic impact associated with the decrease in gene expression, was observed in the injected population of 100 insects (3 independent trials). The same phenomenon was observed when we targeted the 1276 bp part of *SnJHER* (Fig. 1C). However, when we used the dsJHER_1725_, a SnJHERi specific phenotype (described in the next session) was obvious in an average of 5% of the total injected animals (N = 100, three independent trials), (Fig. 1D, Table S2 in File S1). In order to check whether there is a correlation between the used constructs and the efficiency of the RNAi after intra-hemolymph administration in L5d3 larvae, we analyzed the semi-quantitative RT-PCR data in terms of % silenced animals in the total population of the 15 randomly selected individuals (3 independent trials). Our results showed that, the percentage of the total silenced individuals is increased from dsJHER_472_ to dsJHER_1725_. This increase was statistically significant (student’s t-test, p<0.05, 3 biological replicates) comparing the dsJHER_1725_ with the dsJHER_472_ and dsJHER_1276_ constructs, (Table S1 in File S1).

For prepupal stage we injected animals of 6^th^ instar d9. After prepupal JHERi, an average of >90% of the total (N = 100, three independent trials) dsJHER 472 bp/ or 1276 bp/ or 1725 bp-injected animals failed to ecdyse to the next pupal instar and died as larval-pupal intermediates, (Table S3, S4 and S5 in File S1; see next session). Randomly selected individuals 3 days post injection, were subjected to semiquantitative RT-PCR analysis in order to measure the *SnJHER* mRNA levels. All of the randomly selected larval-pupal intermediates were found to contain low *SnJHER* mRNA levels, when compared to the control-untreated ones (Fig. 1E, 1F and 1G). We note that in other lepidopteran insects, maximum silencing effects were observed between 2–4 days post-injection [25], [26].

#### Bacterial administration

For bacterial dsJHER administration we used the same part of the 5′-translated region of *SnJHER* that previously resulted no phenotypic effects (dsJHER_472_) (Fig. 1A). This was expressed in the RNAse III deficient HT115 (DE3) *E. coli* strain. The bacterial administration would be more persistent and prolonged comparing with the hemolymph administration method.

**Figure 2:**
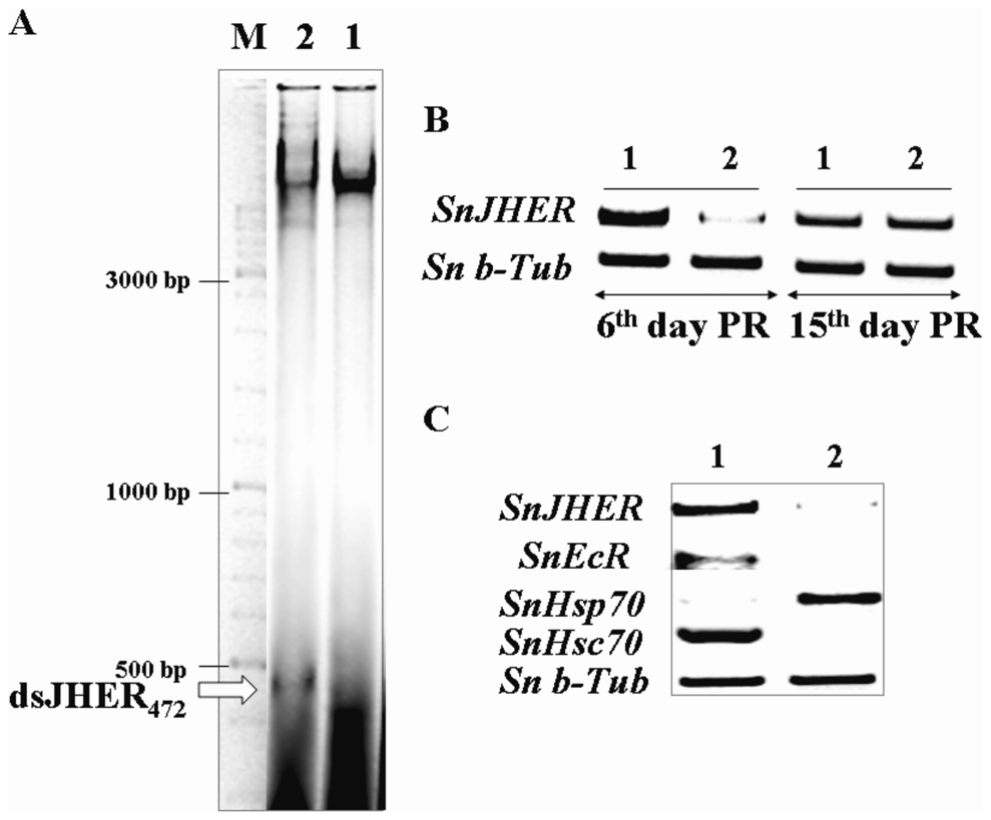
Targeting the *SnJHER* 472 bp part after bacterial administration of dsJHER_472_. **A.** Confirmation of dsJHER_472_ synthesis in IPTG induced HT115 bacteria. Agarose gel electrophoresis of RNA extracted from IPTG induced HT115/L4440 (1) and HT115/pGEM T-easy-JHER_loop_ (2) bacteria followed by RNase-A treatment in high salinity buffer. **B.** Semiquantitative RT-PCR analysis of JHER gene in randomly selected pools of 10 insects recovered from a continuous feeding assay (1^st^→5^h^ instar d0) with the HT115 bacteria (1, 2), 6 and 15 days post recovery (PR). **C.** Semiquantitative RT-PCR analysis of *JHER*, *EcR*, *Hsp70* and *Hsc70* in randomly selected pools of 10 insects from a continuous feeding assay (5^th^→6^th^ instar) with the HT115 bacteria (1, 2), 7 days post feeding. As reference gene it was used the *Sesamia*’s *b-tubulin* gene.

The insects were fed in artificial diet that was supplemented with the dsJHER expressing bacteria. Three bioassays were subsequently performed. In the first bioassay the IPTG induced bacteria were supplied to *S. nonagrioides* larvae from the d0 of the first instar till the d0 of the 5^th^ instar d0 (N = 100, three independent trials). After this period of continuous feeding, the insects were placed to their normal diets and sampled for RT-PCR analysis 6 and 15 days post recovery. Pools of ten randomly selected insects were subjected to RT-PCR analyses. The RT-PCR showed a decrease of *SnJHER* mRNA levels 6 days post recovery (5^th^ instar d6) while the mRNA levels returned to a normal accumulation 15 days post recovery (6^th^ instar d5) (Fig. 2B). Since no phenotypic effect was observed in the first biossay, a second one was performed, in which the induced bacteria were applied to *S. nonagrioides* larvae for a more extended time, representing the entire larval life of the insect (L1d0→L6d9, N = 100, three independent trials). Also in this case a SnJHERi specific phenotype was not observed (data not shown). In a third biossay, the insects were fed with dsRNA expressing bacteria for a shortened period of 7 days, from 5^th^ instar d0 until 5^th^ instar d7 (N = 100, three independent trials). Ten randomly selected individuals were RNA extracted, mixed in one RNA pool and subjected in RT-PCR analyses for semi-quantifying the *SnJHER*, mRNA levels (Fig. 2C). In addition, expression of other developmental genes, *SnHSPs* and *SnEcR*, which were expected to be affected by *SnJHER* knockdown, was also quantified. The sampling day was chosen according to previous reports in which maximum silencing is observed after 7 days of continuous feeding with the dsRNA producing bacteria [22], [27]. It was indeed observed that continuous feeding with dsRNA expressing bacteria resulted in significant knockdown of *SnJHER* mRNA and that silencing of *SnJHER* affected expression of the other developmental genes (Fig. 2C; see further below). Also in this case a SnJHERi specific phenotype was not observed.

#### Baculovirus-mediated RNAi

For baculovirus-mediated RNAi we used the same part of the 5′-translated region of *SnJHER* that previously resulted into no phenotypic effects, either with hemolymph or bacterial mediated administration (dsJHER_472_) (Fig. 1A). The appropriate baculovirus strain was selected according to the supplementary text (Text S1 in File S1). Insects of several developmental stages were injected with 10^7 ^pfu/ml of the BmNPV-BmA::GFP/BmA::JHER_loop_ virus. As control we used a virus expressing double stranded molecules of the luciferase gene (BmNPV/BmA::GFP-BmA::dsLuciferase virus). The infections were carried out at two different developmental stages, 5^th^ instar d3 (larval stage) and 6^th^ instar d9 (prepupal stage) larvae.

The experiments were performed in independent triplicates (three independent trials) of a total amount of 200 insects. Each trial was consisted of at least 60 insects either for control and experimental groups, (Table S6, S7 in File S1). Following infections, the insects were allowed to complete their development recording daily for phenotypic effects suggestive of developmental abnormalities. For RT-PCR analyses insects were sampled 7 days post infection, the day in which the maximum infectivity of the BmNPV virus in terms of GFP expression was observed (Fig. S3).

**Figure 3:**
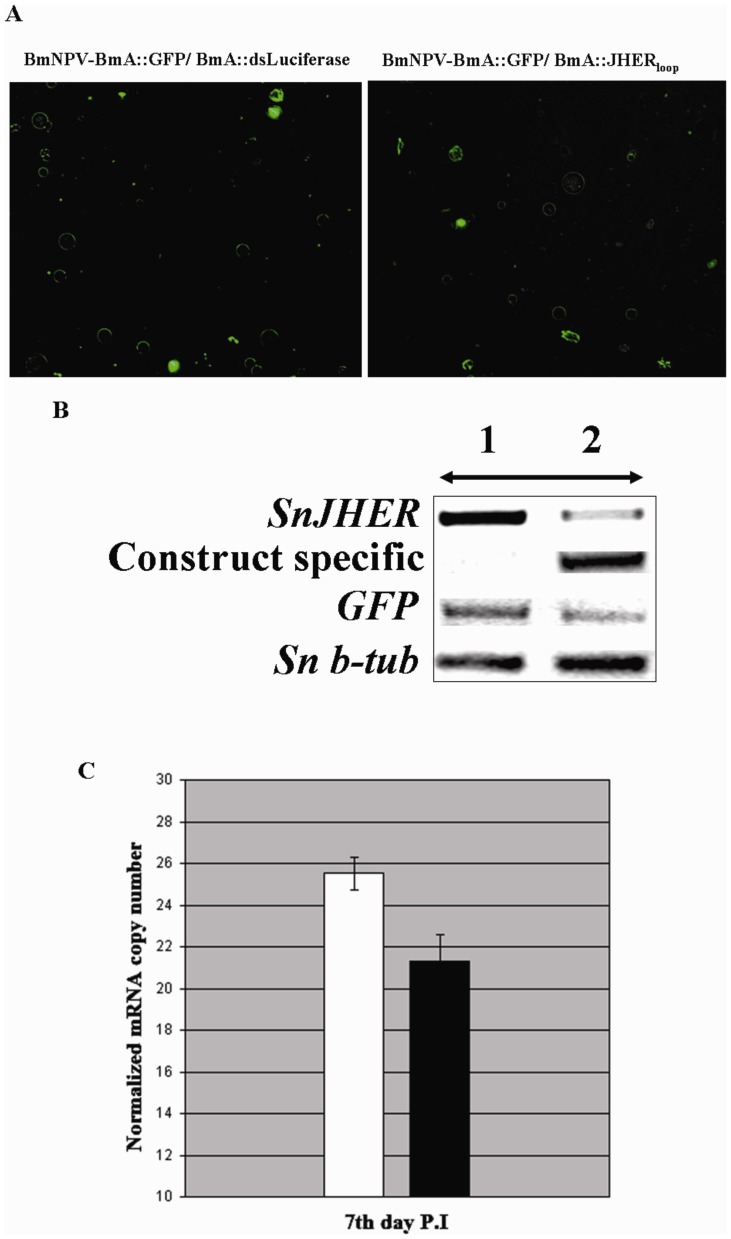
Targeting the *SnJHER* 472 bp part after baculovirus administration of dsJHER_472_. **A.** Fluorescence field images of BmNPV-BmA::GFP/BmA::dsLuciferase and BmNPV-BmA::GFP/BmA::JHER_loop_ infected animals. **B.** Semiquantitative RT-PCR analysis of *SnJHER*, JHER_loop_ and *GFP* gene in randomly selected BmA::GFP/BmA::dsLuciferase (1) and BmNPV-BmA::GFP/BmA::JHER_loop_ infected animals (2), 7 days post infection **C.** Real time RT-PCR analysis of *SnJHER* mRNA levels in 14 randomly selected BmA::GFP/BmA::dsLuciferase (White column) and BmNPV-BmA::GFP/BmA::JHER_loop_ (Black column) infected individuals, 7 days post infection. The bars above the columns indicate the S.E. of the mean of 14 samples with three technical replicates.

Randomly selected SnJHERi positive animals were subjected to semi-quantitative RT-PCR analysis in order to measure the *SnJHER*, GFP and construct specific mRNA levels. In Fig. 3B we present a particular case of an analyzed individual. This analysis showed that the *SnJHER* mRNA levels were specifically decreased, 7 days post infection in the BmNPV-BmA::GFP/BmA::JHER_loop_ infected animals, while the GFP mRNA levels were similar in both BmNPV-BmA::GFP/BmA::dsLuciferase and BmNPV-BmA::GFP/BmA::JHER_loop_ infected larvae (Fig. 3B). The JHER hairpin mRNA was only expressed in the BmNPV-BmA::GFP/BmA::JHER_loop_ infected larvae (Fig. 3B). Moreover, 14 SnJHERi phenotype positive BmNPV-BmA::GFP/BmA::JHER_loop_ infected animals and their BmA::GFP/BmA::dsLuciferase infected controls (5 from trial 1, 4 from trial 2 and 5 from trial 3, Table S6 in File S1) were subjected to quantitative RT-PCR analysis, in order to measure the average silencing levels (Fig. 3C). This analysis showed that the *SnJHER* mRNA levels were decreased by 20% in the BmNPV-BmA::GFP/BmA::JHER_loop_ infected larvae 7 days post infection, when compared to the control BmA::GFP/BmA::dsLuciferase infected ones (Fig. 3C). Fig. 3C represents the relative expression levels of *SnJHER,* normalized to these of b-tubulin gene. The bars indicate the S.E. of the mean of 14 samples with three technical replicates.

### Phenotypic Analysis

#### Hemolymph administration

**Figure 4:**
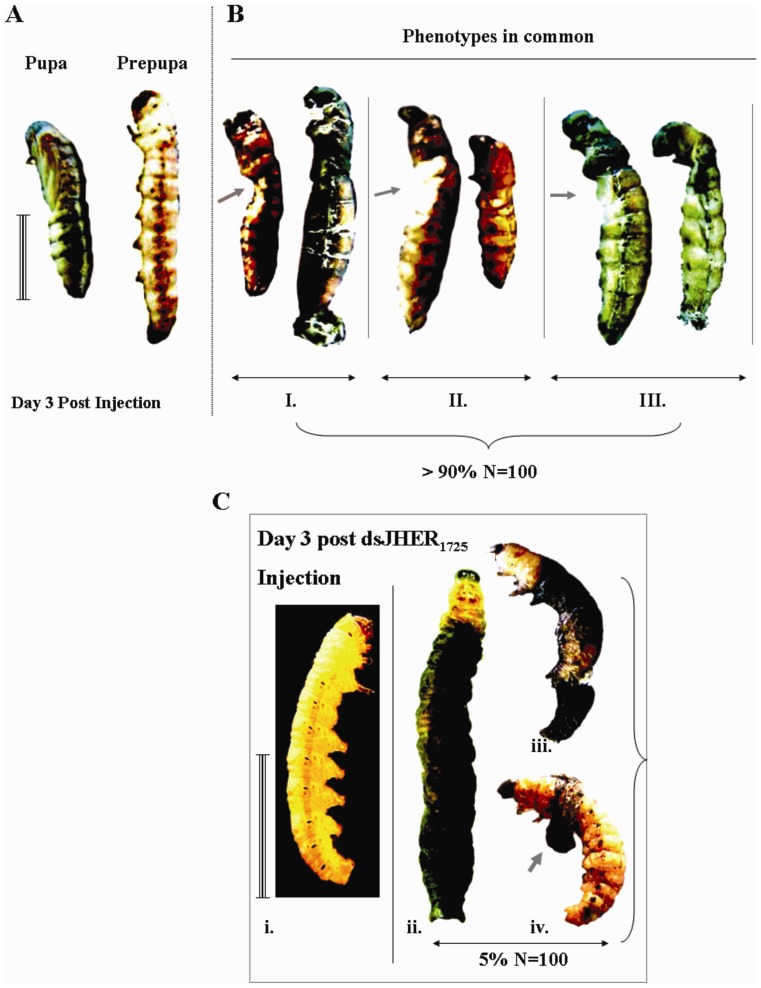
Phenotypic results after hemolymph administration of dsJHERs. **A.** Left: Normal Pupa, Right: Normal 6^th^ instar d9 larva (Prepupa). **B.** Targeting the *SnJHER* 472 bp (BI.) or the *SnJHER* 1276 bp (BII.) or the *SnJHER* 1725 bp (BIII.) part after hemolymph administration of dsJHERs in 6^th^ instar d9 larvae. **BI.** Resulted larval-pupal intermediates after hemolymph administration of dsJHER_472_ in 6^th^ instar d9 larvae, **BII.** Resulted larval-pupal intermediates after hemolymph administration of dsJHER_1276_ in 6^th^ instar d9 larvae, **BIII.** Resulted larval-pupal intermediates after hemolymph administration of dsJHER_1725_ in 6^th^ instar d9 larvae. The arrows indicate similar abnormalities observed in intermediates of all targeted regions. In each case, -I-, -II- or -III- the abnormalities were presented in an average of >90% of the injected animals, 3 days post injection, (N = 100). **C.** Developmental abnormalities of dsJHER_1725_ 5^th^ instar d3 injected larvae. i. Normal larva, ii. Abnormal larva with fused (double) melanized epidermis of the previous instar. iii. Lateral view of abnormal larvae with fused epidermis. iv. The same larva of previous case -iii.-, by removing the melanized epidermis. The arrow indicates the fusion point between the previous and the new epidermal tissues. The newest epidermis is of yellowish colour. Scale bar: 1 cm

Figure 4B shows the phenotypic impact of prepupal hemolymph administration of dsJHER_472_, dsJHER_1276_ and dsJHER_1725_ dsRNAs. Prepupal hemolymph administration of each one of these constructs resulted in larval-pupal intermediates in an average of >90% (three independent trials; Table S3, S4 and S5 in File S1) of the total injected population of N = 100 insects (Fig. 4BI, 4BII and 4BIII). These differences were statistically significant since none of these phenotypic abnormalities were presented in dsL4440_MCS_ injected controls (Student’s t-test, p<0.05; Table S3, S4 and S5 in File S1). On the contrary, in larval hemolymph administration, none of the dsJHER_472_ and dsJHER_1276_ constructs resulted in a SnJHERi specific phenotype, despite the efficient transcriptional silencing of the gene (data not shown). However, administration of SnJHER_1725_ resulted in a SnJHERi specific phenotype in an average of 5% of the total injected (N = 100, three independent trials, Table S2 in File S1) animals (Fig. 4C). These insects were incapable to shed their old cuticles, which were fused with the newest synthesized epidermis dying during the molting procedure (Fig. 4C). This phenotype was obvious in all three independent replicates, and never was presented in the dsL4440_MCS_ injected controls (Student’s t-test, p<0.05; Table S2 in File S1).

#### Baculovirus-mediated RNAi

**Figure 5:**
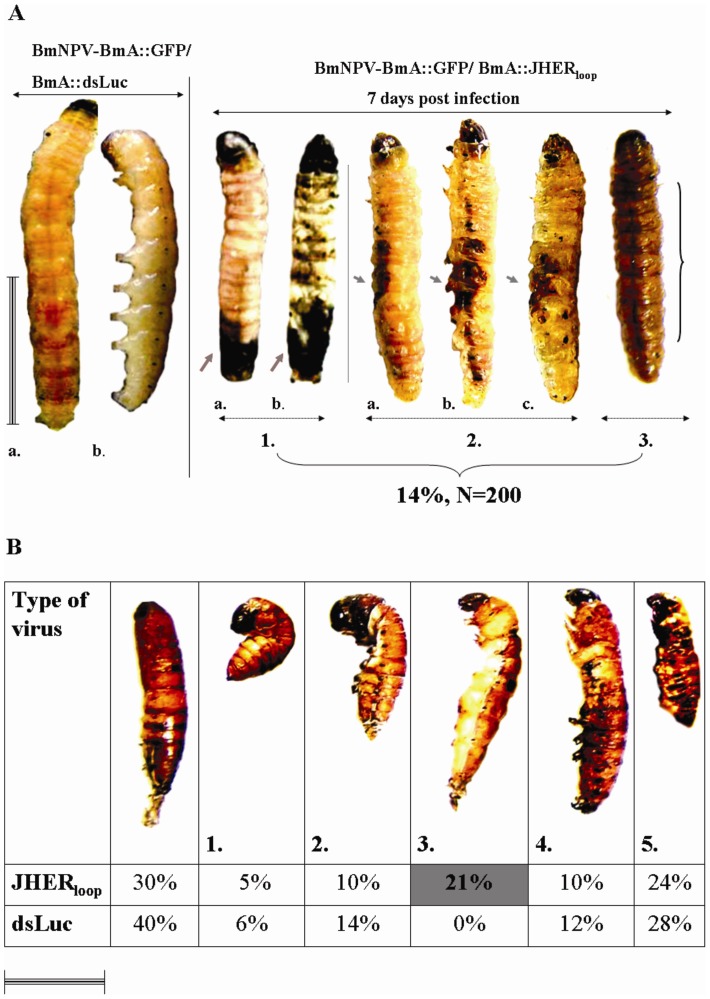
Phenotypic results after baculovirus-mediated dsJHER_472_ administration. **A.** Targeting the *SnJHER* 472 bp part after baculovirus administration of dsJHER_472_ in 5^th^ instar d3 larvae Left. Infection with BmNPV-BmA::GFP/BmA::dsLuciferase virus 7^th^ day post infection (N = 200), a. Dorsal and b. Lateral view of BmNPV-BmA::GFP/BmA::dsLuciferase infected animals. Right. Infection with BmNPV-BmA::GFP/ BmA::JHER_loop_ virus 7^th^ day post infection (N = 200), 1. Type I of developmental abnormalities of BmNPV-BmA::GFP/BmA::JHER_loop_ infected animals, melanized epidermis in the posterior side. a. Dorsal and b. Abdominal view of BmNPV-BmA::GFP/BmA::JHER_loop_ infected animals. 2. Type II of developmental abnormalities of BmNPV-BmA::GFP/BmA::JHER_loop_ infected animals, melanized epidermis in the lateral side. a. Dorsal, b. Lateral and c. Abdominal view of BmNPV-BmA::GFP/BmA::JHER_loop_ infected animals. 3. Type III of developmental abnormalities of BmNPV-BmA::GFP/BmA::JHER_loop_ infected animals, melanized epidermis in the whole body, dorsal view. Types I, II, and III were presented in 14 % of the BmNPV-BmA::GFP/BmA::JHER_loop_ infected animals (N = 200). **B.** Targeting the *SnJHER* 472 bp part after baculovirus administration of dsJHER_472_ in 6^th^ instar d9 larvae. Developmental abnormalities of larval-pupal intermediates shared in both BmNPV-BmA::GFP/BmA::dsLuciferase and BmNPV-BmA::GFP/BmA::JHER_loop_ infected animals (1→5). A. Normal pupa. The type 3., of larval-pupal intermediate that was presented only in BmNPV-BmA::GFP/BmA::JHER_loop_ infected animals. Scale bar: 1 cm

For larval RNAi, similar phenotypes to these of hemolymph administration, were observed in an average of 14% of the total BmNPV-BmA::GFP/BmA::JHER_loop_ injected animals (N = 200, three independent trials, Table S6 in File S1), (Fig. 5A). In prepupal RNAi (6^th^ instar d9 infected larvae), both BmNPV-BmA::GFP/BmA::dsLuciferase and BmNPV-BmA::GFP/BmA::JHER_loop_ infected animals, shared several developmental abnormalities of larval-pupal intermediates (Fig. 5B). These phenotypes were categorized (Fig. 5B 1–5) and only one could be considered as SnJHERi specific (Fig. 5B, case 3; Table S7 in File S1). We considered the phenotypic category 3 as JHERi specific by 2 criteria: a. the larval-pupal intermediates presented whitish larval-like epidermis with melanotic spots, b. the *SnJHER* mRNA levels were lower than the BmA::GFP/BmA::dsLuciferase controls. This category was not presented in the BmA::GFP/BmA::dsLuciferase infected animals and was statistically significant compared with the common larval-pupal intermediates and normal pupae (three trials, Student’s t-test p<0.05, Table S7 in File S1).

**Figure 6:**
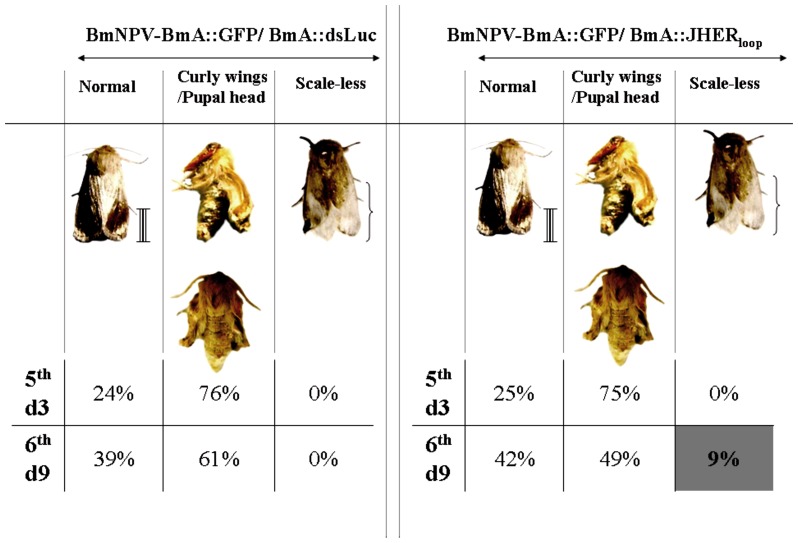
Targeting the *SnJHER* 472 bp part after baculovirus administration of dsJHER_472_. Developmental abnormalities of pupal-adult transition of BmNPV-BmA::GFP/BmA::dsLuciferase and BmNPV-BmA::GFP/BmA::JHER_loop_ infected animals. The scale less phenotype was presented only in the BmNPV-BmA::GFP/BmA::JHER_loop_ infected animals. Scale bar: 1 cm

The surviving pupae were allowed to complete their development. Between the two viruses no significant change in the pupal-adult transition was observed when insects were infected in the 5^th^ instar (Table 1). On the contrary, when insects were infected in the prepupal stage, 26% of the BmNPV-BmA::GFP/BmA::JHER_loop_ infected animals failed to emerge to the adult stage as compared to the 10% of the BmA::GFP/BmA::dsLuciferase infected animals (Table 1, three independent trials, Student’s t-test p<0.05). Moreover, the emerged adults of both cases shared several developmental abnormalities, which could be distinguished in several categories; e.g. normal adults, curly wings/pupal head, scale-less (Fig. 6). The scale-less category was presented only to the BmNPV-BmA::GFP/BmA::JHER_loop_ infected animals in a percentage of 9% of the emerged adults and only on them which were infected in the last larval instar (Fig. 6; Table S8 in File S1, three independent trials, Student’s t-test p<0.05).

**Table 1:** Targeting the *SnJHER* 472 bp part after baculovirus administration of dsJHER_472_.

Type of virus	Trialnumber	Total survived pupaeof larvae infectedin L5d3	Total emerged adultsof larvae infectedin L5d3	% emerged adultsof larvae infectedin L5d3	Total survived pupaeof larvae infectedin L6d9	Total emerged adultsof larvae infectedin L6d9	% emerged adultsof larvae infectedin L5d3
**dsLuciferase**							
	1	66	28	42	27	25	93
	2	66	10	15	26	23	89
	3	68	46	68	25	22	88
**Mean**				42			90
**JHER_loop_**							
	1	58	34	59	19	14	74
	2	58	33	57	20	15	75
	3	56	33	59	23	17	74
**Mean**				58			74
**Statistical significance t-test** **(p<0.05): dsLuciferase versus** **JHER_loop_**				NO			YES

% emerged adults from pupae infected with the BmNPV-BmA::GFP/BmA::dsLuciferase and BmNPV-BmA::GFP/BmA::JHER_loop_ viruses infected as L5d3 or L6d9 larvae. The experiment was replicated 3 times and the mean and the statistical significance of the percentage of the emerged adults between the control and the experimental group (p<0.05, t-test) were calculated.

### SnJHERi Influences the mRNA Synthesis of *S.* *nonagrioides* Ecdysone Receptor, Heat Shock Cognate 70 and Heat Shock Protein 70 Genes

In order to shed light on the potential molecular communication between *SnJHER* and several components of the basal endocrine system of *S. nonagrioides*, including the ecdysone receptor (GenBank: JN572102), heat shock cognate 70 (GenBank: DQ004584) and heat shock protein 70 (GenBank: EU430480) genes, we selected to silence *SnJHER* through bacterial RNAi. This method should cause less stress effects in treated insects, in terms of technical handling (injections versus infections). In that way, any transcriptional alteration in HSP gene expression, will has been caused by the RNAi effect and not the technical stress. Semiquantitative RT-PCR analysis of *SnJHER* mRNA in RNA pools of 10 randomly selected individuals seven days post feeding, showed a decrease in *SnJHER* mRNA levels, suggesting efficient *JHER* silencing (Fig. 2C). Moreover, the mRNA accumulation of *SnHsp70* is increased while the *SnHsc70* and *SnEcR* mRNA accumulation is decreased after *SnJHER* silencing (Fig. 2C).

### Summary of the Results

As we can see in Table 2, hemolymph administration of dsJHER in prepupal stage resulted in a SnJHERi specific phenotype in an average of >90% of the total injected prepupae (N = 100 for each construct used, three independent trials) by any construct used. In contrast to the prepupal, in larval RNAi only injection with the longest dsRNA (dsJHER_1725_) resulted in a SnJHERi specific phenotype in the 5% of the total injected (N = 100 animals, three trials). Bacterial RNAi, on the other hand, resulted in no phenotypic effects, although efficient knockdown was achieved. On the contrary, baculovirus-mediated dsJHER administration resulted in a SnJHERi specific phenotype in an average of 14% of the total infected instead of the 5% of the total dsJHER_1725_ injected animals (three independent trials). Moreover, baculovirus-mediated RNAi complemented the prepupal RNAi through hemolymph administration resulting into useful information regarding the pupal-adult transition.

**Table 2:** Summary of the results after hemolymph, bacterial, baculovirus-mediated dsJHER administration.

Type of administration	Targeted region	Time of administration	Total insects*	Control	Total insects* (Controltreatments)	Specific *SnJHER* silencing	Phenotype
**Injection**	472 bp	5^th^d3	100	dsL4440_MCS_	100	Yes	Absent
	1276 bp	5^th^d3	100	dsL4440_MCS_	100	Yes	Absent
	1725 bp	5^th^d3	100	dsL4440_MCS_	100	Yes	5% of melanized/fused epidermis
	472 bp	6^th^d9	100	dsL4440_MCS_	100	Yes	>90% of larval-pupal intermediates type I.
	1276 bp	6^th^d9	100	dsL4440_MCS_	100	Yes	>90% of larval-pupal intermediates type II.
	1725 bp	6^th^d9	100	dsL4440_MCS_	100	Yes	>90% of larval-pupal intermediates type III.
**Bacterial feeding**	472 bp	1^st^d05^th^d0	100	HT115-L4440	100	Yes	Absent
	472 bp	1^st^d06^th^d9	100	HT115-L4440	100	Yes	Absent
	472 bp	5^th^d05^th^d7	100	HT115-L4440	100	Yes	Absent
**Baculovirus infection**	472 bp	5^th^d3	200	dsLuciferase Virus	200	Yes	14% of melanized/fused epidermis
	472 bp	6^th^d9	200	dsLuciferase Virus	200	Yes	21% of larval-pupal intermediates type 3
	472 bp	Survived pupae from 6^th^d9 infections	78	dsLuciferase Virus	62	Yes	9% of scale-less adults

The asterisk indicates the total amount of insects used, among three independent trials, both for control and experimental groups.

## Discussion

This paper describes the functional characterization of a JHE related gene in the moth *S. nonagrioides*. For this insect, standard reverse or forward genetics techniques have not been established, hindering any attempt for an accurate functional characterization of an experimental gene. For non-model lepidopteran insects, several reverse genetics techniques have been used in the past, with variable efficiencies and successes regarding the obtained results. Ιn order to cover the whole spectrum of the technical difficulties that could result into erroneous experimental conclusions, we selected to use different RNAi approaches. These differed in terms of the dsRNA delivery method including hemolymph, bacterial or baculovirus-mediated. Our combined results shed light on the functional characterization of *SnJHER* and the efficacy of the techniques used for this characterization.

### RNAi Efficiency

In larval RNAi through hemolymph injection, the incapability of dsJHER_472_ and dsJHER_1276_ to develop a SnJHERi specific phenotype, despite efficient silencing of the gene (Fig. 1A, 1B, 1C), could be explained by several reasons related to the efficiency of this method. In a publication, which described the efficiency of RNAi after hemolymph administration in the model organism *Tribolium castaneum*, Sherry et al. [28] found that longer dsRNAs were more effective than shorter ones with respect to both the initial knockdown and the duration of the RNAi effect. This was not due to differences in length *per se*, but to the fact that the longer dsRNA produce a greater variety of siRNAs some of which could be more effective at silencing level than the limited number of siRNAs produced by the shorter dsRNA fragment. Moreover, the size of dsRNA seemed to have a more drastic affect on the duration of RNAi than on the initial RNAi efficiency [28]. Here, we observed almost the same phenomenon, in terms of the efficiency of the hemolymph dsRNA administration to produce a phenotype associated with the *SnJHER* RNAi. While dsJHER_1725_ injection resulted in abnormal larvae in the 5% of the total injected animals (Fig. 4C), dsJHER_472_ and dsJHER_1276_ only caused a reduction of *SnJHER* mRNA levels without producing any SnJHERi specific phenotype (Fig. 1B, 1C). We speculate that dsJHER_1725_ was more effective regarding the duration of the RNAi effect and cell penetration efficiency as happened with *T. castaneum*. In contrast to the larval injections, in the prepupae all constructs were capable of producing a SnJHERi specific phenotype (Fig. 4B). The stage dependent effectiveness of RNAi in terms of phenotype production in other Lepidopteran species has previously reported [29]. In the silkworm, *B. mori* it has been claimed that the stage of early wandering (EW) larvae (prepupal stage) is more sensitive to RNAi [30]. Furthermore, in *Drosophila melanogaster*, larval RNAi through hemolymph dsRNA administration is not effective for many tissues, despite the success of adult injection. This may suggest different tissue specificity at different developmental stages; the basis of the difference between larval and adult tissues is still unknown, but may be due to fundamental developmental differences between tissue types, such as cell ploidy, or due to differences in gene expression required for the uptake and transport of dsRNA [31].

Bacterial administration of dsJHER_472_ had no developmental consequence in *S. nonagrioides* larvae, despite the efficient silencing of the gene, at any treatment tested (Fig. 2B, 2C). This could be explained as previously, by factors that have to deal with the RNAi ineffectiveness, with respect to the initial knockdown, duration of the RNAi effect and cell penetration efficiency.

The phenotype produced by infection with the BmNPV/dsJHER virus was similar with this of the hemolymph administration of dsJHER_1725_ in the 5^th^ larval instar of the insect (Fig. 4C, 5A). The *Autographa californica* nuclear polyhedrosis virus (AcMNPV) and *Bombyx mori* nuclear polyhedrosis virus (BmNPV) encode an ecdysteroid UDP-glucosyltransferase (EGT) gene, which inactivates ecdysone by conjugating the hydroxyl group at C-22 with a sugar [32], [33], [34], [35]. Insects infected with a virus containing the gene encoding EGT do not molt because of a lack of active ecdysone [32], [33], [35]. BmNPV’s infections showed a high blockage of larval-pupal and pupal-adult molt of *S. nonagrioides* in contrast to the AcMNPV’s infections in which the molting arrest was observed in larval instars as well (see Text S1 in File S1). We speculate that BmNPV in contrast to AcMNPV’s EGT is not effective enough to block molting procedure during the larval-larval molts of the corn stalk borer, probably due to its gene expression dynamics or enzyme’s biochemical efficiency; maybe an indirect consequence of the BmNPV’s host incompatibility. Baculovirus administration method may be more effective in terms of the systematic distribution of the expressed dsRNAs. Consequently, baculovirus-mediated RNAi could not be applied directly to functionally characterize genes of the lepidopteran insects since in pupal and adult stages the infection effects mask the potentially produced phenotypes after the loss of function of the experimental gene (Fig. 5B, 6).

### *SnJHER* Function

We have previously shown that *SnJHER* was rhythmically up-regulated close to each molt during *S. nonagrioides* larval development [20]. Additionally, we demonstrated that while the JH analog methoprene does not affect *SnJHER* gene expression, ecdysteroids induce the *SnJHER* mRNA synthesis. Combining these two results, we speculate that *SnJHER* is following the ecdysteroid titer of *S. nonagrioides* in contrast to other conventional JHE genes of several insects in which the JHE mRNA levels are following the JH titer. The findings of the current work complete our previous results suggesting that *SnJHER* is implicated in larval-larval molt of *S. nonagrioides* larvae. Insects of 5^th^ instar d3 injected with 4 µg of dsJHER_1725_ or infected with 50 µl of 10^7 ^pfu/ml of the BmNPV-BmA::GFP/BmA::JHER_loop_ virus presented several developmental abnormalities including a total failure to complete the molting process (Fig. 4C, 5A). Searching the global contemporary literature, there are no similar works in other insect species, in order to compare them with our results. On the contrary, there are many publications describing the application effects of several JH analogs or JHE inhibitors in the development of many insect species including the lepidopterans. In the neuropteran, *Chrysoperla carnea* larvae treated with the juvenile hormone analog fenoxycarb showed two major alterations of pre-imaginal development: i. the inhibition of metamorphosis and ii. the inhibition of cocoon spinning [36]. In this species, metamorphosis was strongly affected by fenoxycarb. Aside from the presence or not of a complete cocoon, a high percentage of larvae did not succeed in metamorphosing to adults. Insects were considered to be affected by inhibition of metamorphosis when: a. they were still alive when the non-affected larvae were already inside a cocoon and b. they continued as larvae, prepupae, pupae or pharate adults, never becoming adults. When mortality occurred in the period when the non-affected larvae had already metamorphosed, this was considered to be a consequence of metamorphosis inhibition, although the exact cause of mortality was unknown. Should *JHER* be a *JHE* conventional gene, we would expect that larval SnJHERi in the corn stalk borer would cause an extension in larval life rather than a blockage in the molting procedure. We conclude that *SnJHER* could be implicated in the ecdysteroid instead of the JH signalling of *S. nonagrioides* by interfering with the molting process.

Our data demonstrated that SnJHERi through hemolymph administration, resulted in a total failure of larval-pupal metamorphosis in >90% of the total injected prepupae by all used constructs (dsJHER_472_, dsJHER_1276_ and dsJHER_1725,_ Fig. 4A). In addition, BmNPV-BmA::GFP/BmA::JHER_loop_ infected prepupae shared several categories of developmental abnormalities of larval-pupal intermediates with the control infected ones, but only one of them could be considered as SnJHERi specific (Fig. 5B, case 3). Previously we have shown that *SnJHER* mRNAs were low in the beginnings of the 5^th^ instar and increased gradually until L5d3, just before the larval ecdysis [20]. In the 6^th^ (last) larval instar, *SnJHER* mRNAs were lower than those of the 5^th^ instar and increased gradually from L6d4 to L6d5, when they peaked; on the next days, the transcripts declined and disappeared [20]. The total physiological impact of SnJHERi in the final larval instar of the corn stalk borer suggests that this gene is important for the larval-pupal transition despite its low expression during the L6. Moreover the low *SnJHER* mRNA levels in L6d9 compared with the high mRNA levels in L5d3 could be the reason for the total observed abnormality in this particular instar after SnJHERi.

Infection of prepupae with the BmNPV-BmA::GFP/BmA::JHER_loop_ virus resulted in a decrease of adult emergence compared with the control treated ones (Table 1). Moreover, some of the BmNPV-BmA::GFP/BmA::JHER_loop_ emerged adults presented a scale-less wing morphology (Fig. 6). The deficiency in pupal-adult transition was also observed in *Spodoptera exigua EcR* silenced animals. In this species silencing of *SeEcR* resulted in a total adult malformation and emergence complications. In *Sesamia* the adult abnormalities which were observed after the silencing of *SnJHER* in the last larval instar indicate that they may be a possible consequence of the indirect downregulation of *SnEcR*, caused by the SnJHERi. However due to the common deficiencies between the control and experimental groups, an indirect effect of the virus infection, it is still extremely difficult to distinguish the JHERi effects in this particular instar with baculovirus-mediated dsRNA administration [37].

Here we also showed that two major components of the ecdysteroid pathway, the *SnEcR* and the *SnHsc70* genes are downregulated after SnJHERi (Fig. 2C). There are numerous studies showing the importance of the ecdysone receptor gene in the control of the molting process. In *D. melanogaster* mutants of the *EcR-B* isoform present predominant time of death between the 1^st^ and 2^nd^ larval stages [38]. Many of the dead *EcR-B* mutants carry a duplicated larval cuticle suggesting that they have arrested during the process of larval molting. The *Drosophila* Hsc70 is required for activation of the EcR/USP heterodimer *in vivo* [39]. The USP polypeptide folds appropriately into a relatively stable configuration that is not further stabilized by chaperones. By contrast, the EcR polypeptide folds into an unstable configuration easily subject to irreversible unfolding or protease degradation. The unstable EcR interacts with appropriate *Drosophila* chaperones including Hsp90 and Hsc70, which stabilize EcR in a configuration appropriate for formation of EcR/USP heterodimers capable of binding EcRE DNA sequences [39]. The phenotypic results of the SnJHERi experiments showed similar molting deficiencies with those of the *EcR-B* mutants of *Drosophila*. Considering the importance of *DmHsc70* in the stabilization of the EcR/USP heterodimer *in vivo*, we conclude that there may be a relation between the downregulation of the *S. nonagrioides EcR* and *Hsc70* genes and the developmental abnormalities observed in the JHER silenced animals. The developmental deficiencies observed after the silencing of *SnJHER* indicate that they may be a possible consequence of the indirect downregulation of *SnEcR* and *SnHsc70* genes, caused by the reduction of the *SnJHER* mRNA levels.

## Conclusion

Our results showed that *SnJHER* presents important biological functions regulating the larval, pupal and adult development. With respect to the high diversity of insect COEs in either substrate specificity or developmental gene expression we speculate that *SnJHER* may possess distinct molecular functions than the conventional *JHE* gene. Further biochemical studies are needed, in terms of substrate specificity and enzyme’s selectivity, in order to shed light on the functional role of this gene in the regulation of the corn stalk borer’s development.

## Materials and Methods

### 

#### Insect rearing and staging of larvae

The insects were obtained from an established laboratory colony of *S. nonagrioides,* maintained at 25 ± 1°C, 55 ± 5% RH and reared on an artificial diet [20], under long day (LD) conditions (16∶8, light:dark). Larvae which were reared under LD conditions completed their larval stage in 6 instars. The age of analyzed larvae within each instar was measured in days after the preceding ecdysis, in respect to physiological markers such as body mass and head capsule width. The nomenclature of stages follows the pattern of designation of the instar followed by the day of the stadium (e.g. L5d2 denotes larvae of the 5^th^ instar, 2 days after ecdysis). Larvae were checked daily for molting. The age of the analyzed larvae within each instar was measured in days after the preceding ecdysis and in respect to physiological markers such as body mass and head capsule width. To obtain synchronously growing animals, newly molted larvae were removed from the colony everyday during the 6^th^–8^th^ hour of photophase. The selected larvae had mean weight and mean head capsule width as follows: 101.3 mg and 1.74 mm (L5d0); 160.4 mg and 2.32 mm (L6d0). In the 9^th^ day of the last instar, larvae transform into prepupae (L6d9) and begin all the necessary physiological and morphological changes in order the metamorphosis to occur.

#### Insect cell growth and maintenance

*Bombyx mori* Bm5 cells [40] were grown in IPL-41 insect cell culture medium, supplemented with 10% fetal bovine serum (Life Technologies), were maintained at 28°C and subcultured weekly.

#### RNA isolation and cDNA synthesis

Total RNA was isolated from larvae and insect cells using TRIzol® reagent (Sigma) according to the supplier’s instructions and stored at −80°C. The isolated RNA was treated with the RNase-free DNAse I (Promega) and 1.5 µg of it, was used as template in first strand cDNA synthesis. The cDNAs were synthesized by priming with the universal poly-thymine primer Oligodt (Table 3), using as reverse transcription enzyme, the Superscript™ II RNase H-Reverse Transcriptase (Invitrogen). In all experiments the RNA was extracted from the whole body tissue of the analyzed animals.

**Table 3:** Primers used in this study.

Primer 5′3′, used as	Name	Sequence	Tm°C
Forward/Reverse	3′F	AGGGACGACCTCATGAAATACTG	59
Reverse	3′R	GACACTAGGATGACGCACTCTTG	57
Reverse	5′R	GCTGACTAAATATTCGGGTCCA	58
Forward	ECRF	AGATTACATTATTAAAGGCGTGCTC	57
Reverse	ECRR	GAGATGCACATGTTGGAGTTCTGC	59
Forward	GFPF	GCTTCTCGTTGGGGTCTTTG	56
Reverse	GFPR	TCCAGGAGCGCACCATCTTC	57
Forward	Hsc70F	CTTCTTCCCTGAGGAAGTTAGC	59
Reverse	Hsc70R	TGTCGTTGGTGATGGTAATCTTG	58
Forward	Hsp70F	GGCTGAGAAGGACGAGTATGAG	59
Reverse	Hsp70R	CAATATGGAAATGCAAGTCTGG	60
Reverse	Oligodt	GTCGACCTCGAG(T17)	
Forward/Reverse	T7	TAATACGACTCACTATAGGG	56
Forward	TubF	GAGCAGTTCACCGCTATGTTC	59
Reverse	TubR	GGTGTGAGTGCTTTAGTTGTCC	58
Forward/Reverse	Wf	AACATGTTACTGTTGCGGAAGC	58

#### Bright field and UV field microscopy

All fluorescence observations were conducted directly on living cells or tissues using a Zeiss Axiovert 25 inverted microscope equipped with a HBO 50 illuminator for incident-light fluorescence excitation and a Zeiss filter set 09 (450–490 nm excitation filter, 510 nm barrier filter).

Quantitative and Semiquantitative RT-PCR analysis

For semiquantitative and quantitative RT-PCR analysis of *SnJHER* (GenBank: EU178813) and semiquantitative RT-PCR analysis of *SnEcR* (GenBank: JN572102), *SnHsc70* (GenBank: DQ004584), *SnHsp70* (GenBank: EU430480), *GFP* and *construct specific* mRNA levels we used the primer sets, 3′F/3′R, ECRF/ECRR, Hsc70F/Hsc70R, Hsp70F/Hsp70R, GFPF/GFPR and Wf/3′F respectively (Table 3). As control, part of the coding region of *S. nonagrioides* b-tubulin gene (GenBank: DQ147771) was amplified by using the primer set TubF/TubR, (Table 3). The RT-PCR products were separated on 1.5% agarose gels. Incorporation of the fluorescent dye SYBR Green Brilliant (Stratagene) into double-stranded PCR products was used to determine the mRNA copy number of *SnJHER*. Standard plasmids were constructed by inserting a fragment from the coding region of *SnJHER* (using the primer set 3′F/3′R, Table 3) or *Sesamia nonagrioides* b-tubulin (using the primer set TubF/TubR, Table 3) into pGEM T-easy vector (Promega). These plasmids were used as template DNA to produce standard curves. Each sample was analyzed in technical triplicates and the means were calculated. The quantity of mRNA levels was normalized with those of b-tubulin.

### Hemolymph Administration of dsJHER

#### dsRNA quantity/ Control treatments

For all experiments we used 4 µg of the *in vitro* synthesized dsRNAs. For control injections we selected an *in vitro* synthesized dsRNA produced by the multiple cloning site of L4440 vector (Addgene), flanked by the T7 promoter sequences. To our knowledge, GFP-based or other “neutral” constructs or just ddH_2_O, which we had previously used as controls in RNAi experiments, resulted in the same effects in terms of semiquantitative/quantitative RT-PCR or phenotypic analysis when compared with these of the L4440’s multiple cloning site (Table S9 in File S1). Previous studies were also underlining that [25]. The probe for RNA synthesis was isolated from L4440’s multiple cloning site of ∼250 bp, by amplifying with the universal T7 primer (Table 3). The amplified fragment flanked by the T7 promoter sequences was used as a template for dsRNA synthesis; T7 RNA polymerase (Fermentas) was allowed to RUN off overnight at 37°C. DNA was removed by DNase treatment (Promega). The dsRNA was then phenol/chloroform extracted, alcohol precipitated overnight and quantified.

#### Targeting the 472, 1276 and 1725 bp part

PCR was performed using cDNA isolated from *S. nonagrioides* larvae fat tissue, by priming with the Wf/5′R and Wf/3′R primer sets, which amplify 472 and 1725 bp respectively, (Table 3). The PCR products were gel extracted and T/A cloning was performed in pGEM T-easy vector (Promega). For pGEM T-easy/JHER_472_ two clones were selected one with SP6T7 and the other with T7SP6 orientation (Fig. S1A). Both clones (sense and antisense) were linearized with SalI (New England Biolabs) and used as templates for RNA synthesis. The 1725 bp fragment was excised from pGEM T-easy/JHER_1725_ with EcoRI (New England Biolabs) and ligated in the EcoRI position of pBIISK- vector (Agilent Technologies). The clone with T3→T7 orientation (pBIISK-/SnJHER_1725T3T7_) was double digested with XhoI/NcoI (New England Biolabs) and the resulting 1276 bp fragment was force ligated into the L4440 vector (L4440/ SnJHER_1276_, Fig. S1B). The L4440/ SnJHER_1276_ plasmid was then linearized with either XhoI or NcoI (New England Biolabs) and used as template for RNA synthesis. For dsJHER_1725_ synthesis the pBIISK-/SnJHER_1725T3T7_ (Fig. S1C) was linearized with either XhoI (New England Biolabs) or XbaI (New England Biolabs). RNA synthesis was performed with T7 or T3 RNA polymerase (Fermentas). Sense and antisense RNA strands were quantified and equal amounts of RNA were mixed and annealed at boiling water for 10 minutes. Hybridization was performed by gradient cooling the boiled mix overnight. Plasmid DNA removed by DNase treatment. The dsRNA was phenol/chloroform extracted, alcohol precipitated overnight and quantified.

### Bacterial Administration of dsJHER

#### Control treatments

For control treatments, we transformed bacteria with the empty L4440 vector. This vector produces dsRNA molecules of ∼250 bp from its multiple cloning site which is surrounded by the T7 promoter sequences. The empty L4440 vector resulted in the same effects, in terms of semiquantitative/ quantitative RT-PCR or phenotypic analysis, when compared with these of GFP-based constructs (Table S1 in File S1). Previous studies were also underlining that [22].

#### Targeting the 472 bp part

PCR was performed using cDNA isolated from *S. nonagrioides* larval fat tissue, by priming with the Wf/5′R and Wf/3′R primer sets. The PCR products were gel extracted and T/A cloning was performed in pGEM T-easy vector. The 1725 bp fragment was excised from pGEM T-easy/JHER_1725_ with EcoRI and ligated in the EcoRI position of pBIISK- vector. The clone with T3→T7 orientation was named as pBIISK-/SnJHERa (Fig. S2A). Furthermore, the pGEM T-easy/JHER_472_ with T7→SP6 orientation was named as pGEM T-easy/SnJHERs (Fig. S2B). Both pBIISK-/SnJHERa and pGEM T-easy/SnJHERs plasmids were double digested with SalI/SacI (New England Biolabs). The SalI/SacI fragment excised from pBIISK-/SnJHERa and was re-cloned to the SalI/SacI digested pGEM T-easy/SnJHERs plasmid. Positive clones were selected after digestion with EcoRI and NotI RE. The resulting plasmid was named as pGEM T-easy/SnJHER_loop_ (Fig. S2C).

HT115 (DE3) competent cells lacking RNase III were prepared using standard CaCl_2_ methodology and were transformed with the pGEM T-easy/SnJHER_loop_ plasmid DNA (Fig. S2C). Single colonies of HT115/pGEM T-easy/SnJHER_loop_ cells were cultured in LB at 37°C with shaking at 220 rpm overnight. The culture was diluted 50-fold in 100 ml LB supplemented with 100 µg/ml ampicillin (Sigma) plus 15 µg/ml tetracycline (Sigma) and cultured at 37°C to OD_600_ = 0.5. Synthesis of T7 polymerase was induced with 0.4 mM IPTG and the bacteria were incubated with shaking for an additional 4 h at 37°C. For feeding experiments, bacteria were centrifuged at 5,000 g for 10 min and resuspended in 0.5 ml of ddH_2_O. In order to check efficient dsRNA expression, total RNAs from the bacterial cells were isolated. Total RNAs from bacterial cells were extracted using TRIzol® reagent (Sigma) according to the supplier’s instructions. The RNA pellets were dissolved in 20 µl of ddH_2_O. In order to remove ssRNAs from the RNA samples 1 µl of 1 mg/ml of RNase-A (Ribonuclease-A from bovine pancreas, Sigma) and NaCl to 0.3 M was added (RNase-A in high salinity buffers selectively digests ssRNAs leaving undigested the dsRNAs, [41]). The reaction occurred for 10 minutes at 37°C. The length and the quality of the produced dsRNAs were confirmed by electrophoresis on 1 % agarose gel (Fig. 2A).

After the confirmation of the dsRNA synthesis, feeding bioassays were followed. 100 ml of the IPTG induced cultures was centrifuged and pellets resuspended in 0.5 ml of ddH_2_O. The artificial diet was cut into different sizes of pellets depending on the instar and the number of feeding larvae. For each 100 *S. nonagrioides* neonates or 1^st^ or 2^nd^ instar larvae, a 10×10×10 mm^3^ pellet was used on which 100 µl of fresh IPTG induced bacteria were applied every 12 hours. For each 100 3^rd^ or 4^th^ instar larvae, 200 µl of fresh IPTG induced bacteria were applied on a 20×20×20 mm^3^ pellet, while for each 50 5^th^ or 6^th^ instar larvae 300 µl of fresh IPTG induced bacteria were applied every 12 hours on a 30×30×30 mm^3^ pellet. The pellets were replaced every 2 days, depending on the remaining undigested material. As control we used IPTG induced HT115 bacteria transformed with the empty L4440 vector.

### Baculovirus-mediated dsRNA Administration

#### Control treatments

As control we used the BmNPV/BmA::GFP-BmA::dsLuciferase virus, a virus expressing double stranded molecules of the reference luciferase gene.

#### dsJHER_loop_ construction

The pGEM T-easy/SnJHER_loop_ plasmid (Fig. S2C) was partially digested by incubating 1 µg of it with 1/10 U of the NotI restriction enzyme (New England Biolabs) for 5 minutes at 37°C. The pFastBac Actin-BGH transfer plasmid (Fig. S2D) was digested with NotI and after dephosphorylation (0.5 U of Alkaline Phosphatase in 50 µl of restriction reaction for 30 min) was ligated with the NotI digested SnJHER_loop_ construct (Fig. S2C). The recombinant plasmid pFastBac Actin-BGH /SnJHER_loop_, was transformed into competent DH10Bac/BmNPV-BmA::GFP cells. Transformed bacteria were selected in LB plates containing 50 µg/ml kanamycin, 7 µg/ml gentamicin, 10 µg/ml tetracycline, 100 µg/ml X-a-gal and 40 µg/ml IPTG (Sigma) after O/N incubation at 37°C. 7 single colonies were picked up and grown in liquid LB with 50 µg/ml kanamycin and 7 µg/ml gentamicin. After O/N incubation, bacmid DNA was extracted and each colony was analyzed in PCR reactions using the Wf primer (Table 3) which amplifies approximately 2.300 bp of the construct. All bacmids were SnJHER_loop_ positive. Bacmids 1, 3, 6 and 7 were used for transfection of Bm5 cells with Escort IV transfection Reagent (Sigma). 7^th^ day post transfection the cells were checked for GFP. Few cells were GFP positive 7^th^ day post transfection (Fig. S4). The supernatants were collected and stored as viral stocks (viral stock 1). 20 µl of each viral stock 1 of BmNPV-BmA::GFP/ BmA::SnJHER_loop_ 1, 3 and 7 were used for infection of Bm5 cells. 7 days post infection the supernatants were collected and stored (viral stock 2). The infected cells were observed for GFP expression. The previous step was repeated for one more time (Fig. S4) and the viral stock 3 was collected. In order to ensure positive transposition of the SnJHER_loop_ construct, PCR was performed either in DNA or cDNA of infected Bm5 cells. The DNA or the cDNA was primed-off with the Wf/3′F primer set (Table 3) which amplifies approximately 750 bp of the construct. All viruses were SnJHER_loop_ positive (data not shown). For *in vivo* assays we selected to use the BmNPV-BmA::GFP/ BmA::SnJHER_loop_ 7 virus (Fig. S4). The viral stock 2 of this virus and a viral stock of the BmNPV-BmA::GFP/ BmA::dsLuciferase virus were used for titration, in order to proceed to the *in vivo* assays. Both viruses were measured to have a titer of approximately 10^7 ^pfu/ml.

#### Biological assays

50 µl of each virus were used for infections. Two different developmental stages were selected in order to perform the infections, the 5^th^ instar d3 (larval stage) and the 6^th^ instar d9 larvae (prepupal stage). Following infections, the insects were allowed to complete their development recording daily the potential developmental abnormalities and phenotypic effects. For RT-PCR analyses we sampled insects 7 days post infection, the day in which we observed the maximum infectivity of the BmNPV virus in terms of GFP expression (Fig. S3).

## Supporting Information

Figure S1 **Schematic representation of vector constructs used in RNAi experiments for hemolymph administration of dsJHER.** **A.** Targeting the 472 bp part: The pGEM T-easy vector/SnJHER_472_ constructs with T7→SP6 and SP6→T7 orientation. **B.** Targeting the 1276 bp part: The L4440/SnJHER_1276_ construct. **C.** Targeting the 1725 bp part: The pBIISK-/SnJHER_1725_ construct with T3→T7 orientation.
(TIF)

Figure S2 **Schematic representation of constructs used in RNAi experiments for bacterial or baculovirus-mediated administration of dsJHER_472_.** A. Construction of pBIISK-/SnJHERa plasmid. B. Construction of pGEM T-Easy/SnJHERs plasmid. C. Construction of the pGEM T-Easy/SnJHER_loop_ plasmid. D. The “transfer” pFast Bac Actin-BGH vector.
(TIF)

Figure S3 **Fluorescence field images of** ***S. nonagrioides*** **5^th^ instar larvae infected with BmNPV-BmA::GFP virus, 7 days PI.** i. Midgut surrounded by fat body tissues (5× focusing), ii. Fat body tissue (5× focusing), iii. Epidermis (5× focusing), iv. Tracheae surrounded by fat body tissues (5× focusing), v. Hemolymph cells (20× focusing), vi. Tracheoles (20× focusing).
(TIF)

Figure S4 **Generation of BmNPV-BmA::GFP/BmA::JHER_loop_ virus.** **A.** Bright/fluorescence field images of transfections performed in Bm5 cells, with randomly selected BmNPV-BmA::GFP/ BmA::JHER_loop_ bacmids, 1, 3, 6 and 7, 7 days post transfection (20× focusing). **B.** Fluorescence field images of Bm5 infected cells with viral stock 2 of viruses BmNPV-BmA::GFP/BmA::JHER_loop_ 1, 3 and 7, 7 days post infection (20× focusing).
(TIF)

File S1 Text S1. Virus strain selection/ Virus infectivity and localization; Table S1. RNAi efficiency, after hemolymph administration in L5d3 larvae; Table S2. Hemolymph administration of dsJHER_1725_ in L5d3 larvae; Table S3. Hemolymph administration of dsJHER_472_ in L6d9 larvae; Table S4. Hemolymph administration of dsJHER_1276_ in L6d9 larvae; Table S5. Hemolymph administration of dsJHER_1725_ in L6d9 larvae; Table S6. Baculovirus-mediated administration of dsJHER_472_ in L5d3 larvae; Table S7. Baculovirus-mediated administration of dsJHER_472_ in L6d9 larvae; Table S8. Baculovirus-mediated administration of dsJHER_472_ in L5d3 and L6d9 larvae; Table S9. Selecting the appropriate control treatment for hemolymph dsRNA administration.
(DOC)
