# Supplementary figures and images for: Functional Characterization of a Juvenile Hormone Esterase Related Gene in the Moth Sesamia nonagrioides through RNA Interference
Source: PLoS One. 2013 Sep 11;8(9):e73834. doi: 10.1371/journal.pone.0073834 (PMC3770702; doi:10.1371/journal.pone.0073834)

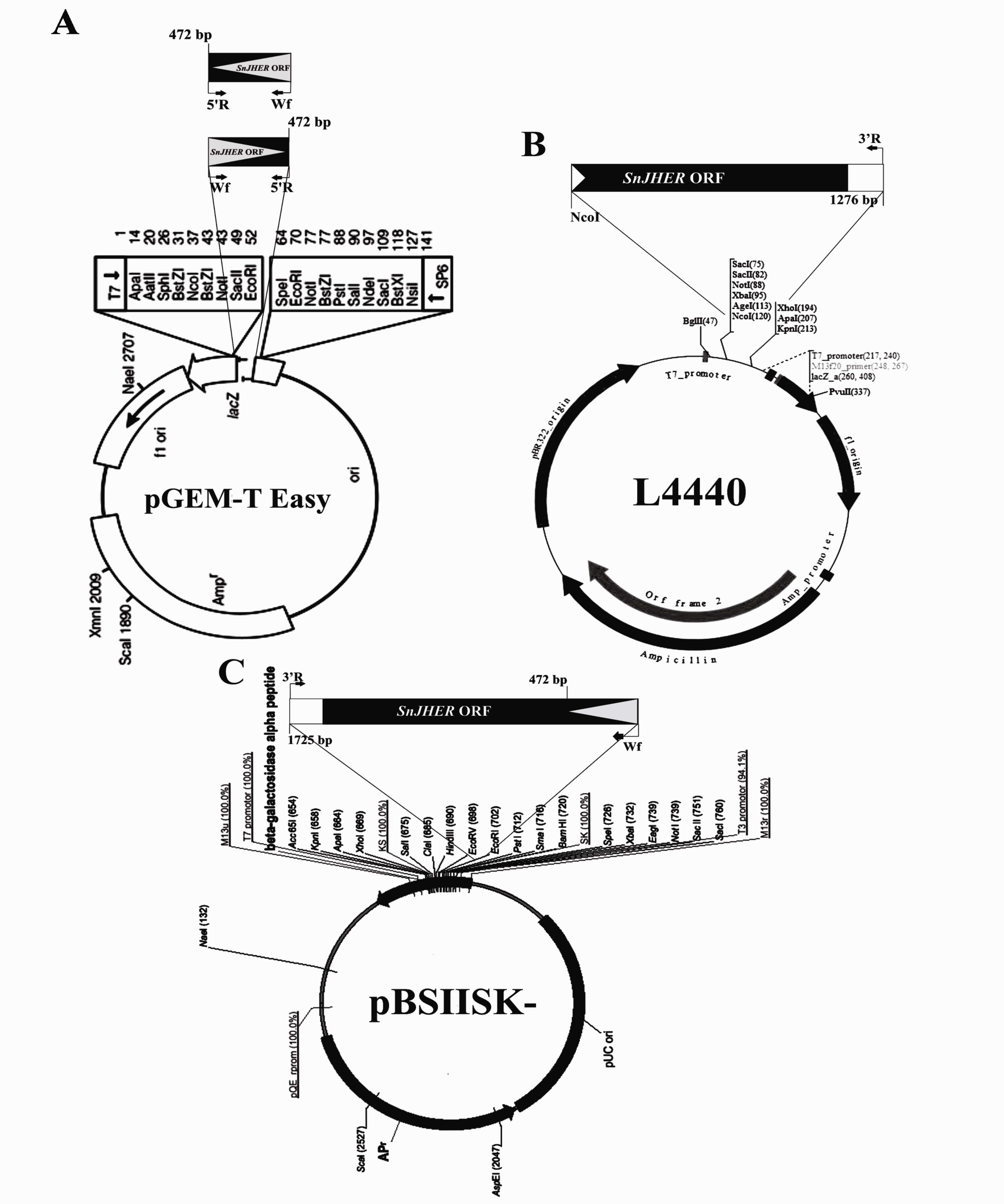

Supplement: Figure S1 — Schematic representation of vector constructs used in RNAi experiments for hemolymph administration of dsJHER. A. Targeting the 472 bp part: The pGEM T-easy vector/SnJHER472 constructs with T7→SP6 and SP6→T7 orientation. B. Targeting the 1276 bp part: The L4440/SnJHER1276 construct. C. Targeting the 1725 bp part: The pBIISK-/SnJHER1725 construct with T3→T7 orientation. (TIF) [file pone.0073834.s001.tif]

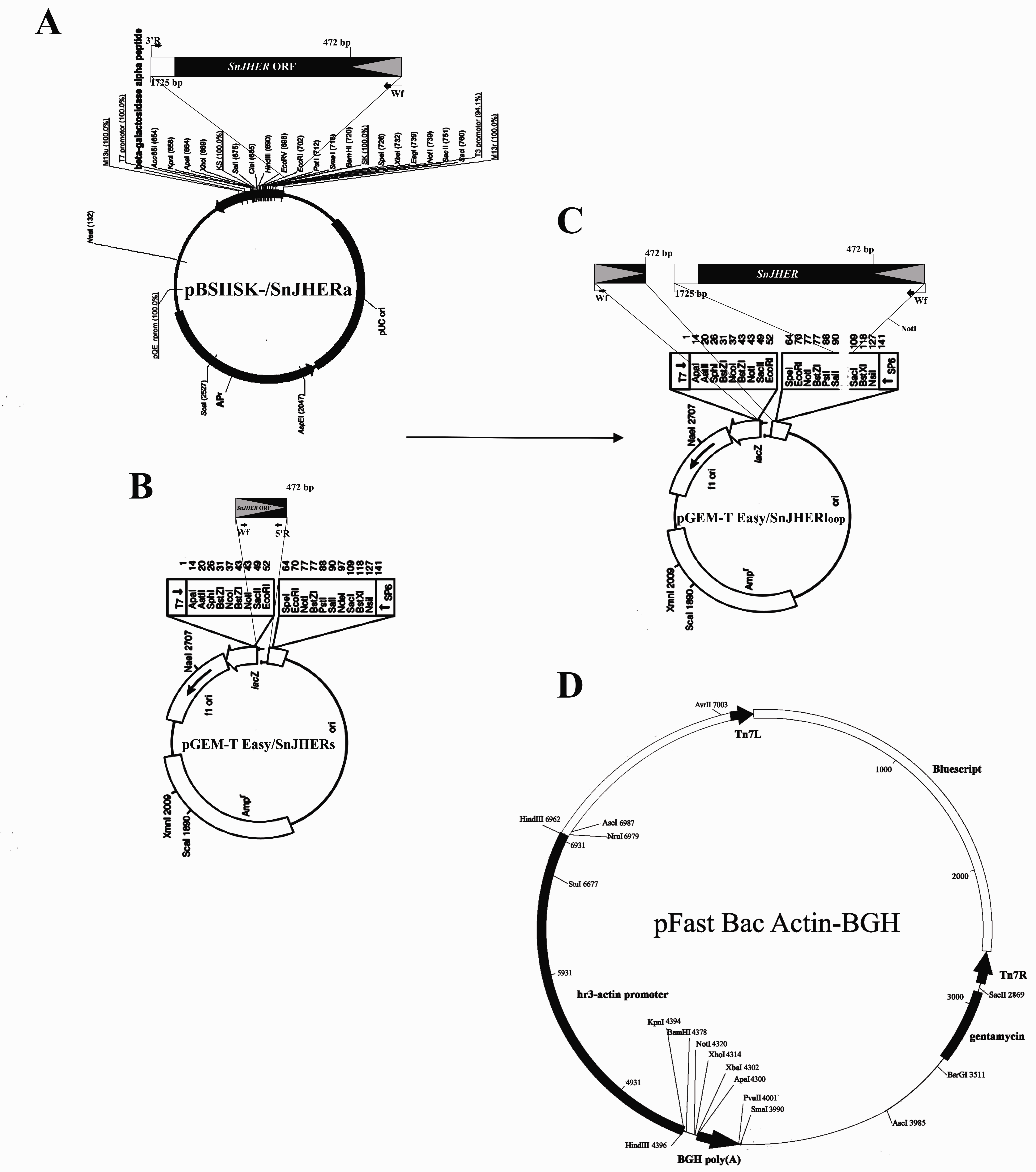

Supplement: Figure S2 — Schematic representation of constructs used in RNAi experiments for bacterial or baculovirus-mediated administration of dsJHER472. A. Construction of pBIISK-/SnJHERa plasmid. B. Construction of pGEM T-Easy/SnJHERs plasmid. C. Construction of the pGEM T-Easy/SnJHERloop plasmid. D. The “transfer” pFast Bac Actin-BGH vector. (TIF) [file pone.0073834.s002.tif]

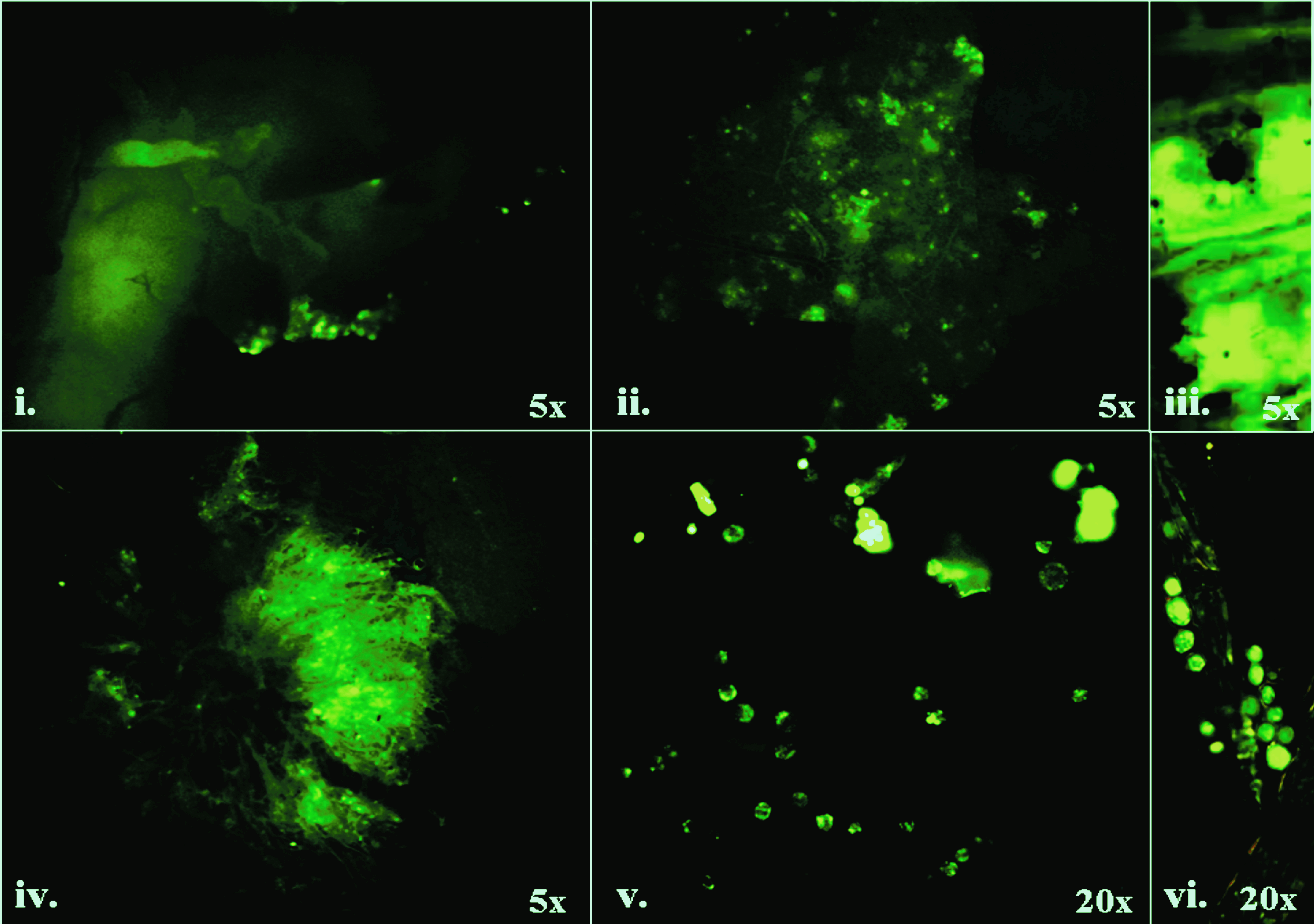

Supplement: Figure S3 — Fluorescence field images of S. nonagrioides 5th instar larvae infected with BmNPV-BmA::GFP virus, 7 days PI. i. Midgut surrounded by fat body tissues (5× focusing), ii. Fat body tissue (5× focusing), iii. Epidermis (5× focusing), iv. Tracheae surrounded by fat body tissues (5× focusing), v. Hemolymph cells (20× focusing), vi. Tracheoles (20× focusing). (TIF) [file pone.0073834.s003.tif]

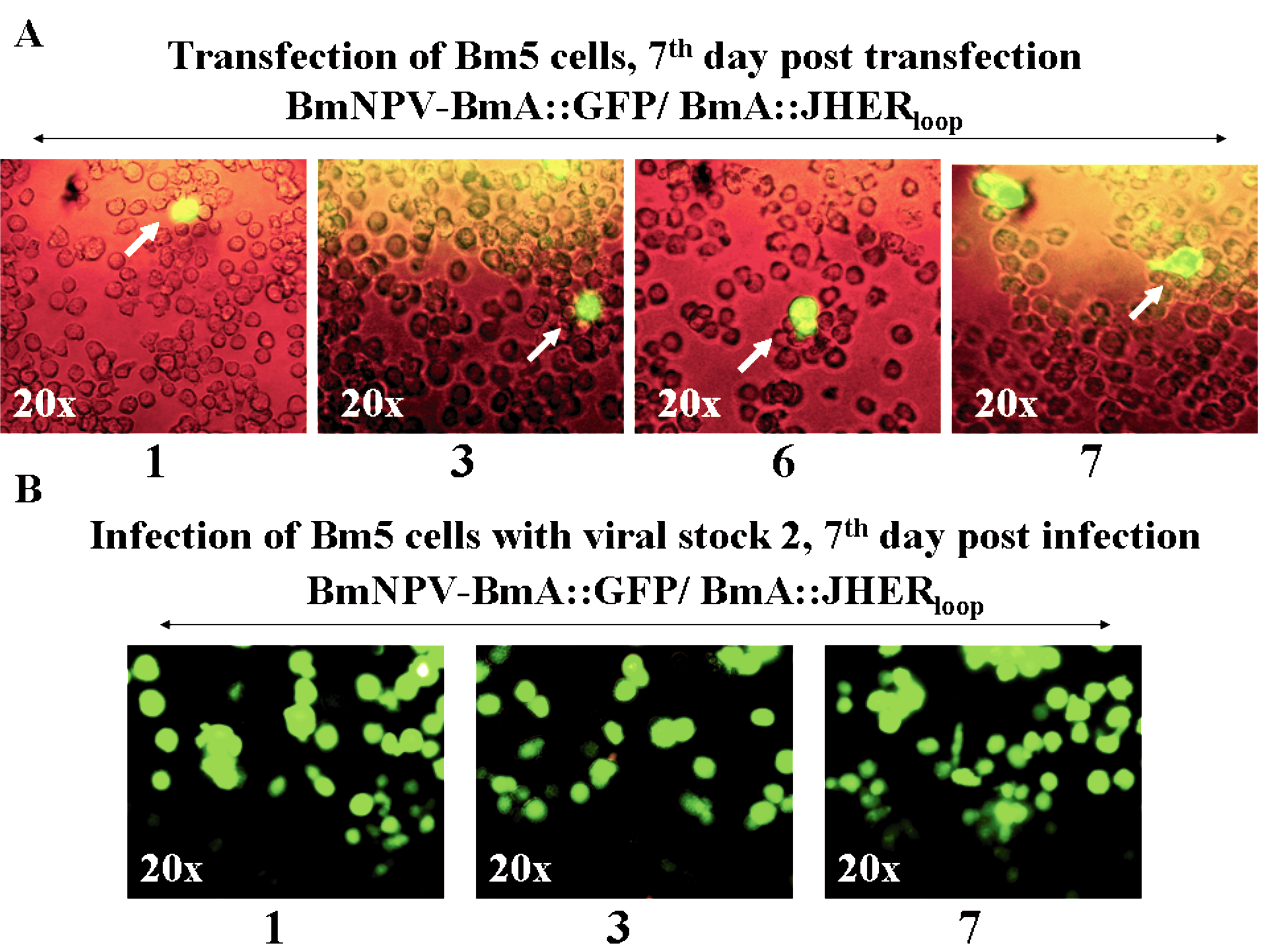

Supplement: Figure S4 — Generation of BmNPV-BmA::GFP/BmA::JHERloop virus. A. Bright/fluorescence field images of transfections performed in Bm5 cells, with randomly selected BmNPV-BmA::GFP/ BmA::JHERloop bacmids, 1, 3, 6 and 7, 7 days post transfection (20× focusing). B. Fluorescence field images of Bm5 infected cells with viral stock 2 of viruses BmNPV-BmA::GFP/BmA::JHERloop 1, 3 and 7, 7 days post infection (20× focusing). (TIF) [file pone.0073834.s004.tif]
