# Supplementary material for: Functional Characterization of a Juvenile Hormone Esterase Related Gene in the Moth Sesamia nonagrioides through RNA Interference
Source: PLoS One. 2013 Sep 11;8(9):e73834. doi: 10.1371/journal.pone.0073834 (PMC3770702; doi:10.1371/journal.pone.0073834)
Supplement: File S1 — Text S1. Virus strain selection/ Virus infectivity and localization; Table S1. RNAi efficiency, after hemolymph administration in L5d3 larvae; Table S2. Hemolymph administration of dsJHER1725 in L5d3 larvae; Table S3. Hemolymph administration of dsJHER472 in L6d9 larvae; Table S4. Hemolymph administration of dsJHER1276 in L6d9 larvae; Table S5. Hemolymph administration of dsJHER1725 in L6d9 larvae; Table S6. Baculovirus-mediated administration of dsJHER472 in L5d3 larvae; Table S7. Baculovirus-mediated administration of dsJHER472 in L6d9 larvae; Table S8. Baculovirus-mediated administration of dsJHER472 in L5d3 and L6d9 larvae; Table S9. Selecting the appropriate control treatment for hemolymph dsRNA administration. (DOC) [file pone.0073834.s005.doc]

**Supplementary text and tables**

**Text S1.**

**Virus strain selection/ Virus infectivity and localization:** The baculoviruses *Bombyx mori* nucleopolyhedrovirus (BmNPV) and *Autographa californica* multinucleocapsid nucleopolyhedrovirus (AcMNPV) share about 90% identity at the genomic level but they have non-overlapping host range and show a high degree of host specificity. In order to assess which kind of virus is ideal for infection of *S. nonagrioides* larvae we performed several bioassays with these two baculovirus strains. Both viruses were pre-transformed with a GFP cassette controlled by the Actin promoter of *Bombyx mori*. The first experiment was performed with the AcMNPV virus. Insects of several larval stages were injected with 10^7^ pfu/ml of the AcMNPV-BmA::GFP virus. Mortality rates, developmental abnormalities and their phenotypic effects were recorded, following infections. The AcMNPV virus caused significant mortality rates in almost 90% of the total injected animals (data not shown). These animals presented all typical symptoms of polyhedrosis. In contrast the survived animals, presented several developmental abnormalities, failing to complete normally their developmental cycle (data not shown). Infected animals were dissected in order to check for GFP expression in their tissues. In contrast to the AcMNPV, the BmNPV infection caused no significant mortality to the injected animals. All infected larvae were able to proceed to their developmental stages and none of them presented any sign of the polyhedrosis (data not shown). Infected animals were dissected in order to check for GFP expression in their tissues. In all experiments using both viral strains, maximum GFP levels were recorded 7 days post infection. Moreover, both viruses were located mostly in the fat body tissue, in the hemolymph, in the epidermal cells and tracheoles of the infected animals (Fig S3). In contrast to the larval infections, when insects were infected with either the AcMNPV-BmA::GFP or the BmNPV-BmA::GFP virus, during the prepupal stage (6^th^ instar d9), they were unable to complete normally their larval-pupal transition and most of them died as larval-pupal intermediates (data not shown). Moreover, the surviving pupae ecdysed to abnormal adults with fused pupal tissues and curly wings (data not shown).

**Table S1.** **RNAi efficiency, after hemolymph administration in L5d3 larvae.** % silenced animals among the randomly selected individuals for RT-PCR analyses. The asterisk represents the statistical significance between the dsJHER_1725_/dsJHER_1276_ and dsJHER_472_ constructs (p<0.05, t-test). Each treatment was replicated 3 times and the mean was calculated.

| **L5d3 injections** | **Trial** | **Number of insects** | **Number of randomly selected insects subjected in RT-PCR analyses** | **Number of randomly selected insects with lower *SnJHER* levels than the dsL4440_MCS_ injected controls** | **Percentage of randomly selected insects with lower *SnJHER* levels than the dsL4440_MCS_ injected controls** |
| --- | --- | --- | --- | --- | --- |
| **dsJHER_472_** | | | | | |
|  | **1** | 32 | 15 | 3 | 20 % |
|  | **2** | 31 | 15 | 3 | 20 % |
|  | **3** | 37 | 15 | 1 | 7 % |
| **Mean** |  | | | | **16 %** |
| **dsJHER_1276_** | | | | | |
|  | **1** | 35 | 15 | 3 | 20 % |
|  | **2** | 31 | 15 | 3 | 20 % |
|  | **3** | 34 | 15 | 2 | 13 % |
| **Mean** |  | | | | **18 %** |
| **dsJHER_1725_** | | | | | |
|  | **1** | 33 | 15 | 7 | 47 % |
|  | **2** | 32 | 15 | 4 | 27 % |
|  | **3** | 35 | 15 | 8 | 53 % |
| **Mean** |  | | | | **42 %*** |

**Table S2. Hemolymph administration of dsJHER_1725_ in L5d3 larvae.** Phenotypic results of dsJHER_1725_ in L5d3 larvae. The experiment was replicated 3 times and the mean and the statistical significance of the percentage of the phenotypic effect between the control and the experimental group (p<0.05, t-test) were calculated.

| **Intra-hemolymph administration**  **of dsJHER_1725_ in L5d3 larvae** | **Trial number** | **Total dsL4440_MCS_**  **injected** | **Total larvae with fused epidermis of dsL4440_MCS_**  **injected** | **% larvae with melanized/fused epidermis of dsL4440_MCS_**  **injected** | **Total dsJHER_1725_**  **injected** | **Total larvae with fused epidermis of dsJHER_1725_**  **injected** | **% larvae with melanized/fused epidermis of dsJHER_1725_**  **injected** |
| --- | --- | --- | --- | --- | --- | --- | --- |
|  | **1** | 32 | 0 | 0 | 33 | 2 | 6 |
|  | **2** | 32 | 0 | 0 | 32 | 1 | 3 |
|  | **3** | 36 | 0 | 0 | 35 | 2 | 6 |
| **Mean** |  | | | **0** |  | | **5** |
| **Statistical significance**  **t-test (p<0.05):**  **Control versus**  **experimental group** |  | | | | | | **YES** |

**Table S3. Hemolymph administration of dsJHER_472_ in L6d9 larvae.** Phenotypic results of dsJHER_472_ in L6d9 larvae. The experiment was replicated 3 times and the mean and the statistical significance of the percentage of the phenotypic effect between the control and the experimental group (p<0.05, t-test) were calculated.

| **Intra-hemolymph administration in L6d9 larvae** | **Trial number** | **Total dsL4440MCS**  **injected** | **Total larval-pupal intermediates of dsL4440MCS**  **injected** | **% larval-pupal**  **intermediates of dsL4440MCS**  **injected** | **Total dsJHER_472_**  **injected** | **Total larval-pupal intermediates of dsJHER_472_**  **injected** | **% larval-pupal**  **intermediates of dsJHER_472_**  **injected** |
| --- | --- | --- | --- | --- | --- | --- | --- |
|  | **1** | 32 | 0 | 0 | 33 | 30 | 91 |
|  | **2** | 33 | 0 | 0 | 34 | 32 | 94 |
|  | **3** | 35 | 0 | 0 | 33 | 31 | 94 |
| **Mean** |  | | | **0** |  | | **93** |
| **Statistical significance**  **t-test (p<0.05):**  **Control versus**  **experimental group** |  | | | | | | **YES** |

**Table S4. Hemolymph administration of dsJHER_1276_ in L6d9 larvae.** Phenotypic results of dsJHER_1276_ in L6d9 larvae. The experiment was replicated 3 times and the mean and the statistical significance of the percentage of the phenotypic effect between the control and the experimental group (p<0.05, t-test) were calculated.

| **Intra-hemolymph administration in L6d9 larvae** | **Trial number** | **Total dsL4440MCS**  **injected** | **Total larval-pupal intermediates of dsL4440MCS**  **injected** | **% larval-pupal**  **intermediates of dsL4440MCS**  **injected** | **Total dsJHER_1276_**  **injected** | **Total larval-pupal intermediates of dsJHER_1276_**  **injected** | **% larval-pupal**  **intermediates of dsJHER_1276_**  **injected** |
| --- | --- | --- | --- | --- | --- | --- | --- |
|  | **1** | 34 | 0 | 0 | 32 | 28 | 88 |
|  | **2** | 33 | 0 | 0 | 35 | 33 | 94 |
|  | **3** | 33 | 0 | 0 | 33 | 31 | 94 |
| **Mean** |  | | | **0** |  | | **92** |
| **Statistical significance**  **t-test (p<0.05):**  **Control versus**  **experimental group** |  | | | | | | **YES** |

**Table S5. Hemolymph administration of dsJHER_1725_ in L6d9 larvae.** Phenotypic results of dsJHER_1725_ in L6d9 larvae. The experiment was replicated 3 times and the mean and the statistical significance of the percentage of the phenotypic effect between the control and the experimental group (p<0.05, t-test) were calculated.

| **Intra-hemolymph administration in L6d9 larvae** | **Trial number** | **Total dsL4440MCS**  **injected** | **Total larval-pupal intermediates of dsL4440MCS**  **injected** | **% larval-pupal**  **intermediates of dsL4440MCS**  **injected** | **Total dsJHER_1725_**  **injected** | **Total larval-pupal intermediates of dsJHER_1725_**  **injected** | **% larval-pupal**  **intermediates of dsJHER_1725_**  **injected** |
| --- | --- | --- | --- | --- | --- | --- | --- |
|  | **1** | 31 | 0 | 0 | 33 | 31 | 94 |
|  | **2** | 35 | 0 | 0 | 34 | 33 | 97 |
|  | **3** | 34 | 0 | 0 | 33 | 30 | 91 |
| **Mean** |  | | | **0** |  | | **94** |
| **Statistical significance**  **t-test (p<0.05):**  **Control versus**  **experimental group** |  | | | | | | **YES** |

**Table S6. Baculovirus-mediated administration of dsJHER_472_ in L5d3 larvae.** Phenotypic results of BmNPV-BmA::GFP/BmA::JHER_loop_ infection in L5d3 larvae. The experiment was replicated 3 times and the mean and the statistical significance of the percentage of the phenotypic effect between the control and the experimental group (p<0.05, t-test) were calculated.

| **Baculovirus-mediated dsJHER472 administration**  **in L5d3 larvae** | **Trial number** | **Total dsLuciferase infected** | **Total larvae with melanized/fused epidermis of**  **dsLuciferase infected** | **% larvae with melanized/fused epidermis of**  **dsLuciferase infected** | **Total**  **JHERloop**  **infected** | **Total larvae with melanized/fused epidermis of JHERloop**  **infected** | **% larvae with melanized/fused epidermis of JHERloop**  **infected** |
| --- | --- | --- | --- | --- | --- | --- | --- |
|  | **1** | 66 | 0 | 0 | 68 | 10 | 15 |
|  | **2** | 66 | 0 | 0 | 66 | 8 | 12 |
|  | **3** | 68 | 0 | 0 | 66 | 10 | 15 |
| **Mean** |  | | | **0** |  | | **14** |
| **Statistical significance**  **t-test (p<0.05):**  **Control versus**  **experimental group** |  | | | | | | **YES** |

**Table S7. Baculovirus-mediated administration of dsJHER_472_ in L6d9 larvae.** Phenotypic results of BmNPV-BmA::GFP/BmA::JHER_loop_ and BmA::GFP/BmA::dsLuciferase viruses infections in L6d9 larvae. The experiment was replicated 3 times and the mean and the statistical significance of the percentage of the phenotypic effect between the control and the experimental group (p<0.05, t-test) were calculated. The asterisk represents the statistical significance (p<0.05), between the phenotypic category 3 and the control group. Numbers in grey boxes represent the 5 phenotypic categories of larval-pupal intermediates observed in both infections.

| **Baculovirus-mediated dsJHER472 administration**  **in L6d9 larvae** | **Trial number** | **Total dsLuciferase infected** | **Total larval-pupal intermediates of dsLuciferase infected animals,**  **Type:** | | | | | **% larval-pupal intermediates of dsLuciferase infected animals,**  **Type:** | | | | | **Total**  **JHERloop**  **infected** | **Total larval-pupal intermediates of JHERloop infected animals,**  **Type:** | | | | | **% larval-pupal intermediates of JHER_loop_ infected animals,**  **Type:** | | | | |
| --- | --- | --- | --- | --- | --- | --- | --- | --- | --- | --- | --- | --- | --- | --- | --- | --- | --- | --- | --- | --- | --- | --- | --- |
|  | | | **1** | **2** | **3** | **4** | **5** | **1** | **2** | **3** | **4** | **5** |  | **1** | **2** | **3** | **4** | **5** | **1** | **2** | **3** | **4** | **5** |
|  | **1** | 65 | 4 | 9 | 0 | 7 | 18 | 6 | 14 | 0 | 11 | 28 | 69 | 4 | 7 | 14 | 8 | 17 | 6 | 10 | 20 | 12 | 25 |
|  | **2** | 67 | 4 | 10 | 0 | 8 | 19 | 6 | 15 | 0 | 12 | 28 | 65 | 3 | 7 | 15 | 6 | 14 | 5 | 11 | 23 | 9 | 22 |
|  | **3** | 68 | 6 | 9 | 0 | 9 | 19 | 6 | 13 | 0 | 13 | 28 | 66 | 2 | 6 | 13 | 6 | 16 | 3 | 9 | 20 | 9 | 24 |
| **Mean** |  | | 5 | 9 | **0** | 8 | 19 | 6 | 14 | **0** | 12 | 28 |  | 3 | 7 | **14** | 7 | 16 | 5 | 10 | **21** | 10 | 24 |
| **Statistical significance**  **t-test (p<0.05):**  **Control versus**  **experimental group** |  | |  | |  |  | |  | |  |  | |  |  | | ***** |  | |  | | ***** |  | |

**Table S8. Baculovirus-mediated administration of dsJHER_472_ in L5d3 and L6d9 larvae.** Phenotypic results of adults emerged from pupae infected with the BmNPV-BmA::GFP/BmA::JHER_loop_ and BmA::GFP/BmA::dsLuciferase viruses as L5d3 or L6d9 larvae. The experiment was replicated 3 times and the mean and the statistical significance of the percentage of the phenotypic effect between the control and the experimental group (p<0.05, t-test) were calculated.

| **Type of virus** | **Trial number** | **Total survived pupae of larvae infected in L5d3** | **Total emerged adults infected in L5d3** | **Emerged adults**  **Phenotypic category,**  **Number, (Percentage):** | | | **Total survived pupae of larvae infected in L6d9** | **Total emerged adults infected in L6d9** | **Emerged adults**  **Phenotypic category,**  **Number, (Percentage):** | | |
| --- | --- | --- | --- | --- | --- | --- | --- | --- | --- | --- | --- |
|  |  |  |  | **Normal** | **Curly wings/Pupal head** | **Scale-less** |  |  | **Normal** | **Curly wings/Pupal head** | **Scale-less** |
| **dsLuciferase** | | | | | | | | | | | |
|  | **1** | 66 | 28 | 7 (25%) | 21 (75%) | 0 (0%) | 27 | 25 | 9 (36%) | 16 (64%) | 0 (0%) |
|  | **2** | 66 | 10 | 2 (20%) | 8 (80%) | 0 (0%) | 26 | 23 | 10 (44%) | 13 (56%) | 0 (0%) |
|  | **3** | 68 | 46 | 13 (28%) | 33 (72%) | 0 (0%) | 25 | 22 | 8 (36%) | 14 (64%) | 0 (0%) |
| **Mean** |  | | | **7 (24%)** | **21 (76%)** | **0 (0%)** |  | | **9 (39%)** | **14 (61%)** | **0 (0%)** |
| **JHERloop** | | | | | | | | | | | |
|  | **1** | 58 | 34 | 8 (24%) | 26 (76%) | 0 (0%) | 19 | 14 | 4 (29%) | 8 (57%) | 2 (14%) |
|  | **2** | 58 | 33 | 9 (27%) | 24 (73%) | 0 (0%) | 20 | 15 | 6 (40%) | 8 (53%) | 1 (7%) |
|  | **3** | 56 | 33 | 8 (24%) | 25 (76%) | 0 (0%) | 23 | 17 | 10 (59%) | 6 (35%) | 1 (6%) |
| **Mean** |  | | | **8 (25%)** | **25 (75%)** | **0 (0%)** |  | | **7 (42%)** | **7 (49%)** | **1 (9%)** |
| **Statistical significance**  **t-test (p<0.05):**  **Control versus**  **experimental group (Scale-less category)** |  | | |  |  |  |  | |  |  | **YES** |

**Table S9. Selecting the appropriate control treatment for hemolymph dsRNA administration.**

| **Type of administration** | **Time of administration** | **N (Insects)** | **Treatment** | **Sampling for RT-PCR analysis** | **Non specific *SnJHER* silencing** | **Non specific phenotype** |
| --- | --- | --- | --- | --- | --- | --- |
| **Injection** | **5thd3 or 6thd9** | 30 | dsL4440_MCS_ | Randomly selected individual/Pool of 10 randomly selected individuals | Absent | Absent |
|  |  | 30 | dsGFP | Randomly selected individual/Pool of 10 randomly selected individuals | Absent | Absent |
|  |  | 30 | Injection buffer | Randomly selected individual/Pool of 10 randomly selected individuals | Absent | Absent |
| **Bacterial feeding** | **1std0🡪6thd9** | 30 | HT115 | Randomly selected individual/Pool of 10 randomly selected individuals | Absent | Absent |
|  |  | 30 | HT115/L4440 | Randomly selected individual/Pool of 10 randomly selected individuals | Absent | Absent |
|  |  | 30 | HT115/L4440-GFP | Randomly selected individual/Pool of 10 randomly selected individuals | Absent | Absent |
